# To Kill a Macrophage: Targeted Strategies to Eliminate Macrophage Reservoirs of HIV

**DOI:** 10.3390/v18030347

**Published:** 2026-03-12

**Authors:** Laura Rikard-Bell, Morgane Brunton-O’Sullivan, Sushama Telwatte, Anthony Jaworowski, Anna C. Hearps

**Affiliations:** 1 Life Sciences Discipline, Burnet Institute, Melbourne, VIC 3004, Australia; laura.rikard-bell@burnet.edu.au (L.R.-B.); morgane.brunton@burnet.edu.au (M.B.-O.); anthony.jaworowski@honorary.burnet.edu.au (A.J.); 2 School of Translational Medicine, Monash University, Melbourne, VIC 3004, Australia; 3 Department of Infectious Diseases, The University of Melbourne at The Peter Doherty Institute for Infection and Immunity, Melbourne, VIC 3000, Australia; sushama.telwatte@unimelb.edu.au; 4 Department of Infectious Diseases, Monash University, Melbourne, VIC 3004, Australia; 5 School of Health and Biomedical Sciences, RMIT University, Bundoora, VIC 3083, Australia

**Keywords:** HIV cure, macrophages, cell death, apoptosis, HIV reservoir, HIV latency

## Abstract

Persistent HIV reservoirs in long-lived macrophages pose a unique and formidable challenge to achieving HIV cure. HIV-infected macrophages are more resistant than CD4+ T cells to both virus- and immune-mediated death pathways including apoptosis, facilitating their persistence in tissue sanctuary sites and potential to contribute to viral rebound upon therapy cessation. This resistance is driven by HIV-induced modulation of both intrinsic and extrinsic apoptotic pathways, alongside survival mechanisms including autophagy. In this review, we examine the biological mechanisms promoting macrophage survival and explore novel translational strategies aimed at subverting this resistance. Crucially, we highlight the methodological limitations hindering progress, including the scarcity of robust in vitro macrophage models, the influence of culture conditions, and physiological relevance to macrophages in vivo. We emphasise that a macrophage-inclusive approach, incorporating improved pre-clinical models and developing clinical measurements to quantify the reservoir in human tissue, is essential to successfully eliminate this distinct reservoir and advance toward sustained ART-free remission.

## 1. Introduction

The persistence of HIV reservoirs within latently infected cells despite antiretroviral therapy (ART) remains the main barrier to HIV cure [1,2,3]. While the majority of the HIV reservoir in blood resides in CD4+ T cells, peripheral blood monocytes can also harbour HIV [4,5,6,7] with an estimated 40% of people with HIV (PWH) on ART found to contain intact proviral DNA within blood monocytes [8]. There is extensive evidence of HIV infection of myeloid cells in tissue (reviewed comprehensively by Wong et al. and Veenhuis et al. [9,10]) with viral reservoirs persisting in myeloid cells in lungs, brain, seminal vesicles, and urethra despite ART [8,11,12,13,14,15,16,17,18,19,20,21]. Myeloid reservoirs have been shown to contribute to viral rebound after cessation of ART in humans [22] as well as mouse models [23] of HIV. Despite this, cure strategies to date have largely overlooked the myeloid reservoir. It remains unclear whether macrophage reservoirs persisting in PWH are truly latent or whether low-level viral transcription and translation may occur. This is relevant not only for viral recrudescence but also for the pathogenesis of comorbidities in PWH, as HIV-infected macrophages may contribute to disease pathology in tissues such as the brain [24,25] and lung [26]. Hence, strategies to eliminate this reservoir are important for not only HIV cure but also to improve the healthy lifespan of people with HIV. Due to their distinct biology and location in diverse anatomical locations, HIV-infected macrophages represent unique challenges for elimination. Furthermore, they appear to resist cell death, hence strategies to overcome this resistance may provide an effective approach for elimination. Across HIV elimination strategies, latency reversal is likely to be a critical precursor for reservoir elimination. While latency reactivation may differ substantially between macrophages and CD4+ T cells [27,28,29], a detailed discussion of these differences is beyond the scope of this review. This review focuses on macrophage biology and host–viral interactions that promote the survival of HIV-infected macrophages, explores novel translational approaches to eliminate this reservoir, and emphasises the importance of developing methods to evaluate cure interventions targeting macrophages in humans.

## 2. Macrophage HIV Reservoirs Pose Unique Challenges

Macrophages are a unique and formidable target for HIV cure due to their long lifespan [30,31], heterogeneity, location in tissue sites, and abundance in immunological sanctuary sites like the brain and testes. These features make the macrophage reservoir a significant barrier to cure, but clinically relevant for the pursuit of ART-free viral control.

### 2.1. Heterogeneity and Anatomical Location

Macrophages exist as a heterogenous population shaped by their ontogeny and tissue microenvironment, resulting in diverse phenotypes and functions. They are present in virtually all tissues, including the brain, heart, gut-associated lymphoid tissue (GALT), lung, liver, spleen, and bone [32]. Tissue-resident macrophages are derived from yolk-sac progenitors and foetal liver and are capable of self-renewal, whereas monocyte-derived macrophages (MDM) originate from circulating bone marrow-derived monocytes. The relative proportions of tissue- and monocyte-derived macrophages varies between different tissue locations and is further influenced by factors such as inflammation [33,34,35]. Many tissue sites harbour multiple subsets of macrophages from distinct progenitor sources including yolk-sac-derived microglia and blood monocyte-derived perivascular macrophages in the brain [36] as well as yolk-sac/foetal-liver-derived alveolar macrophages and blood monocyte-derived interstitial macrophages in the lung [37,38,39,40]. An additional layer of heterogeneity is provided by the ability of macrophages to adopt different functional phenotypes known as polarisation states [41]. In HIV, primary infection has been demonstrated to skew macrophages in urethral tissue towards an intermediary macrophage polarisation state and away from traditional M1 or M2 phenotypes [16]. While HIV reservoirs exist in diverse tissue macrophage types, the influence of macrophage heterogeneity and ontogeny on the formation and maintenance of macrophage reservoirs is currently unknown. These anatomical niches pose specific challenges for cure (reviewed by Ferreira et al. [42]), as they can limit immune access and treatment delivery.

### 2.2. Distinct Infection Mechanisms and Cellular Compartmentalisation in Macrophages

The importance of macrophages as HIV reservoirs has been controversial, due to previous data from macaque studies demonstrating HIV can be detected in macrophages following engulfment or phagocytosis of infected CD4+ T cells [43,44]. However, more recent evidence of integrated HIV DNA and HIV RNA in macrophages has convincingly demonstrated their ability to be productively infected (reviewed in [10,45]). Macrophages can be infected by cell-free HIV or through cell-to-cell transmission. Additionally, HIV can be taken up by macrophages via non-traditional HIV entry receptors involving lectin-mediated interactions, such as by Siglec-1 [46]. Within HIV-infected macrophages, nascent virions can accumulate within intracellular vesicles known as virus-containing compartments (VCCs) [47,48]. While these VCCs remain connected to the plasma membrane by narrow microchannels, they can enable virions to be shielded from HIV-specific neutralising antibodies [48] and potentially immune-mediated killing, allowing HIV to survive in these compartments for weeks [49]. In addition to virus budding from the cell surface, viral particles contained in VCCs can be released upon stimulation or cell death. Thus, this unique feature of HIV-infected macrophages can provide a source of replication-competent virus that is hidden from immune surveillance and is an important consideration for targeting HIV reservoirs in these cells.

### 2.3. Resistance to Death

A fundamental characteristic of HIV-infected macrophages is their increased resistance to cell death, the mechanisms of which are discussed in detail below. The elimination of HIV-infected macrophages following latency reversal will require therapeutic strategies that specifically target the underlying host–viral mechanisms that promote macrophage survival.

## 3. Why HIV-Infected Macrophages Do Not Die: HIV-Induced Alterations to Cell Survival Pathways in Macrophages

### 3.1. HIV-Infected Macrophage Resistance to Immune-Mediated Clearance

HIV-infected macrophages are less susceptible to cytotoxic CD8+ T-cell- and natural killer (NK) cell-mediated killing [50,51], consistent with findings from simian immunodeficiency virus (SIV) models [52,53]. This resistance leads to inefficient elimination by immune cells which, if translated in vivo, is likely to enhance persistence. Currently, little is known about the mechanisms associated with resistance to immune-mediated killing of HIV-infected macrophages. The budding of HIV into VCCs may be a mechanism that limits exposure of HIV antigens such as the envelope (Env) protein on the cell surface, and potentially protects macrophages from immune-mediated recognition and killing [51]. Additionally, antibodies targeting specific Env epitopes exhibit differential binding to HIV-infected macrophages as compared to CD4+ T cells [54], which may influence the efficacy of macrophage targeting through antibody-dependent cellular cytotoxicity and other antibody-mediated mechanisms.

Typically, cytotoxic T lymphocytes (CTLs) [55] andNK cells [56] are able to kill virus-infected target cells via the extrinsic pathway and through Fas ligand (FasL) and Fas receptor (FasR) interactions (see Section 3.2.2). However, HIV-1 accessory proteins including Nef down-modulate major histocompatibility complex class I (MHC-I) from the surface of virus-infected cells, including in primary human macrophages [57] to evade CTL-mediated immunity. In theory, this downregulation should make these cells targets for NK cell-mediated cytotoxicity, however (as stated above) in vitro HIV-infected macrophages can resist efficient NK cell-mediated killing. The increased resistance of HIV-infected macrophages to both CTL and NK cell-mediated killing is associated with a heightened inflammatory response from both cytotoxic effector cells and macrophage targets [50,51], which may contribute to the pathogenesis of HIV and associated comorbidities [58]. Further work is required to fully elucidate the mechanisms underpinning this resistance to immune-mediated killing to inform the development of immune-mediated strategies to eliminate HIV-infected macrophages.

### 3.2. HIV-Infected Macrophage Resistance to Virus-Mediated Death

HIV-infected cells, as well as bystander T cells, may undergo programmed or lytic cell death induced by viral replication [59,60]. However, macrophages have evolved distinct mechanisms to resist virus-induced cell death, enabling them to persist as long-lived reservoirs (reviewed in Le Douce et al. [61]). Apoptosis is a key cell death pathway for both virus- and immune-mediated clearance of HIV-infected cells. In the context of HIV cure, targeting apoptosis provides a mechanism to eliminate HIV-infected cells while importantly avoiding inflammatory responses, which is especially crucial in sanctuary sites like the brain. Hence, this review focusses on the evidence for HIV-induced resistance to apoptotic cell death in macrophages.

#### 3.2.1. Evidence for HIV-Mediated Resistance to Apoptosis in Macrophages

Upon differentiation from monocytes, macrophages have been reported to acquire an anti-apoptotic signature [62,63,64]. This intrinsic resistance, alongside the slow replication of HIV in macrophages [65,66], and their ability to sustain long-term HIV infection without significant cell loss [67] is consistent with observations of resistance to apoptosis during HIV infection [68,69]. Macrophages are known to undergo activation-induced cell death, which, alongside apoptosis, is a fundamental host mechanism for resolving infection and limiting inflammation [70]. The impact of HIV infection on apoptosis in monocytes is less clear, with in vitro and SIV models demonstrating enhanced apoptosis of monocytes in response to infection [71], while other reports show monocytes from viraemic PWH exhibit an anti-apoptosis profile [72]. The relative extent to which intrinsic myeloid cell survival mechanisms and specific effects of HIV infection and viral proteins contribute to apoptosis resistance is unclear but has implications for HIV cure strategies.

Despite the long-held dogma that HIV-infected macrophages are resistant to apoptosis, robust in vitro evidence, particularly using primary human-derived cells, remains scarce. Several early mechanistic studies of HIV-infected macrophages did not include definitive functional cell death assays [73,74]. Initial support for this resistance phenotype came from observations that HIV-infected MDM cultures resisted apoptosis following Tumour necrosis factor-related apoptosis-inducing ligand (TRAIL) [68] or lipopolysaccharide (LPS)/cycloheximide (CHX) [75] stimulation. In vivo evidence further supported this concept, demonstrating reduced apoptosis in microglia from post-mortem brain tissue of HIV-seropositive donors compared to seronegative controls [76]. The first clear in vitro demonstration of primary HIV-infected macrophage protection from apoptosis emerged a decade later [77,78,79], with apoptosis resistant profiles observed in macrophage cell lines [64,79,80]. Furthermore, HIV viral proteins such as Tat, Gp120, and Vpr have been consistently implicated in conferring this resistance phenotype [64,75,77,78,80]. However, not all studies report impaired apoptosis in HIV-infected macrophages, with some studies finding no change [81] or even enhanced [82] apoptosis. These key findings are shown in Table 1 and summarised below.

A major limitation of this work is that almost all studies examine bulk infected cultures, making it impossible to distinguish whether observed resistance reflects truly infected cells or impacts on uninfected bystanders. To date, only two studies—by Boliar et al. [81] and Caballero et al. [83]—have directly compared HIV+ (p24+) macrophages with bystander cells and parallel uninfected cultures; further studies with this design are required to fully determine the extent and mechanism of apoptosis resistance in HIV-infected and bystander cells.

**Table 1:** Evidence for resistance to death in HIV-infected macrophage and implicated viral mechanisms.

HIV Exposure (Tropism)	Cell Type	Culture Supplements	Impact on Viability (Marker) ^1^	Mechanism (Measurement) ^2^	Ref.
***Whole virus exposure***					
BaL (R5)	MDM	Human serum + M-CSF	NA	↑ **BCL-2**, **BCL-XL**, ↓ *BAD*, *BAX* (protein)	Guillemard 2004 [73]
LAI (X4)	MDM	Human serum + M-CSF	↓ TRAIL-induced apoptosis (DNA fragmentation assay)	↓ *TRAIL death receptor* (protein) ↑ **MCL-1**, **BFL-1** (mRNA) ↑ **cIAP1**, **cIAP2**, **XIAP** (protein)	Swingler 2007 [68]
BaL (R5)	MDM	Not described	↓ LPS+CHX-induced cell death (DNA viability stain)	NA	Chugh 2007 [75]
NL4-3 (X4)	Microglial cell line (HMC3)	Bovine serum	↓ LPS+CHX-induced cell death (DNA viability stain)	NA	Chugh 2007 [75]
SG3 with transmitter founder Env	MDM	Bovine serum + M-CSF	NA	↓ TRAIL decoy receptors (protein) No Δ *TRAIL death receptors*, *FasR* (protein)	Zhu 2011 [74]
ADA (R5)	MDM	Human serum + M-CSF	↑ apoptosis in p24+ vs. uninfected cells (TUNEL assay)	↑ *BIM* (protein) and mitochondrial localisation No Δ **BCL-2**, **MCL-1**, *BAK*, *BAX*, caspase activation (protein)	Castellano 2017 [82]
Not described	MDM	Human serum	NA	↑ TREM-1 (mRNA, protein)	Yuan 2017 [80]
Transmitter founder virus (R5)	Macrophage cell line (THP-1)	PMA	↓ apoptosis in HIV+ THP-1 macrophages vs. HIV+ THP-1 monocytes (Annexin-V, DNA viability stain)	↑ **MCL-1**, **BCL-2**, **BCL-XL** (protein)	Xue 2017 [79]
BaL (R5)	MDM	Bovine serum + M-CSF	↓ apoptosis (ssDNA)	↑ TREM-1, **BCL-2**, **BCL-XL,** ↓ *BAD*, *BAK* (protein) ↑ *BIM* translocation to mitochondria ↑ apoptosis with TREM-1 siRNA	Campbell 2019 [77]
Pseudotyped BaL	MDM	Human serum + M-CSF	No Δ apoptosis in HIV+ vs. bystander (cell viability dye, caspase-3/7 protein)	↑ anti-apoptotic long noncoding RNA (mRNA)	Boliar 2019 [81]
BaL (R5)	MDM	Human serum + M-CSF	NA	↑ TREM-1 (protein)	Hyun 2019 [84]
IIIB (X4)	Kupffer cells	Bovine serum	NA	↑ TREM-1 (protein, mRNA)	Hyun 2019 [84]
BaL (R5)	MDM	Bovine serum + M-CSF	NA	↑ **XIAP**, **BIRC2** (protein)	Campbell 2020 [86]
CS204 (R5X4)	MDM	Bovine serum + M-CSF	↑ apoptosis with **cIAP1**, **cIAP2** siRNA in HIV+ vs. bystander cells (Annexin-V, DNA viability stain)	NA	Caballero 2021 [83]
BaL(R5)	Monocyte-derived microglia	M-CSF, GM-CSF, NGFβ, CCL2, and IL-34	↓ apoptosis (ssDNA, LDH assay)	↑ **BCL-2**, TREM-1 (protein) ↑ apoptosis with TREM-1 siRNA	Campbell 2023 [78]
***Viral protein exposure***					
Tat	Microglial cell line (HMC3)	Bovine serum	↓ LPS+CHX-induced cell death (DNA viability stain)	Activation of the PI3K/AKT pathway	Chugh 2007 [75]
Vpr	Macrophage cell line (THP-1)	PMA	↓ apoptosis (Annexin-V, DNA viability stain)	No Δ cIAP1, BCL-2 (protein) ↑ apoptosis with cIAP1, cIAP2 siRNA (but not BCL-XL and MCL-1)	Busca 2012 [64]
Vpr	MDM	Bovine serum + M-CSF	No Δ in viability (Annexin-V, DNA viability stain)	No Δ cIAP1, BCL-2 (protein)	Busca 2012 [64]
Tat, Gp120	Mouse macrophage cell line (Raw264.7)	Bovine serum	NA	↑ TREM-1 (mRNA, protein)	Yuan 2017 [80]
Tat, Gp120	Mouse macrophage cell line (Raw264.7) (TREM-1 KD)	Bovine serum	↑ apoptosis (TUNEL assay)	↑ caspase 3 ↓ BCL-2 (protein)	Yuan 2017 [80]
Tat, Gp120	Mouse bone marrow-derived macrophage	Bovine serum + M-CSF	NA	TREM-1 KO ↑ caspase 3 vs. WT cells (protein)	Yuan 2017 [80]
Tat, Gp120, RNA40	MDM	Bovine serum + M-CSF	↓ apoptosis (ssDNA)	↑ TREM-1, BCL-2, BCL-XL (protein)	Campbell 2019 [77]
Env, Gp120, Pro, Rev	MDM	Human serum + M-CSF	NA	↑ TREM-1 (receptor expression)	Hyun 2019 [84]
Tat	Monocyte-derived microglia	M-CSF, GM-CSF, NGFβ, CCL2, and IL-34	↓ apoptosis (ssDNA, LDH assay)	↑ **BCL-2**, **BCL-XL**, TREM-1 (protein)	Campbell 2023 [78]
***Ex vivo***					
Post-mortem brain tissue [CD68+ macrophages/microglia] (PWH *n* = 9; uninfected *n* = 10)			↓ apoptosis (TUNEL assay)	NA	Cosenza 2004 [76]
Post-mortem brain tissue [macrophage/microglia] (PWH on ART, *n* = 5; uninfected *n* = 4)			NA	↑ *BIM* [mitochondria localisation] (protein)	Castellano 2017 [82]
CD68+ PBMCs (PWH *n* = 11; uninfected = 3)			NA	↑ TREM-1 (receptor expression)	Hyun 2019 [84]

Abbreviations: NA: not assessed; TUNEL: Terminal deoxynucleotidyl transferase (TdT)-mediated U dUTP Nick-End Labelling; PMA: Phorbol Myristate Acetate; NGFβ: nerve growth factor beta; CCL2: Chemokine (C-C motif) Ligand 2; IL-34: Interleukin-34; LDH: Lactate Dehydrogenase; PI3K: phosphoinositide-3-kinase; AKT: protein kinase B; KD: knock down; KO: knock out; WT: wild type. ↑: increased expression, ↓: decreased expression, No Δ: no change. ^1^ Only studies that directly assessed viability of macrophages in the presence and absence of HIV infection were included. Bulk HIV-infected cultures were compared to uninfected cultures, unless otherwise stated. HIV was the only stimulus unless otherwise stated. ^2^ Parameter measured is provided in brackets. *Pro-apoptotic molecules* are listed above in *italic* and **anti-apoptotic molecules** are listed in **bold**.

#### 3.2.2. Viral Mechanisms Implicated in Inhibiting Apoptosis in Macrophages

**Figure 1:**
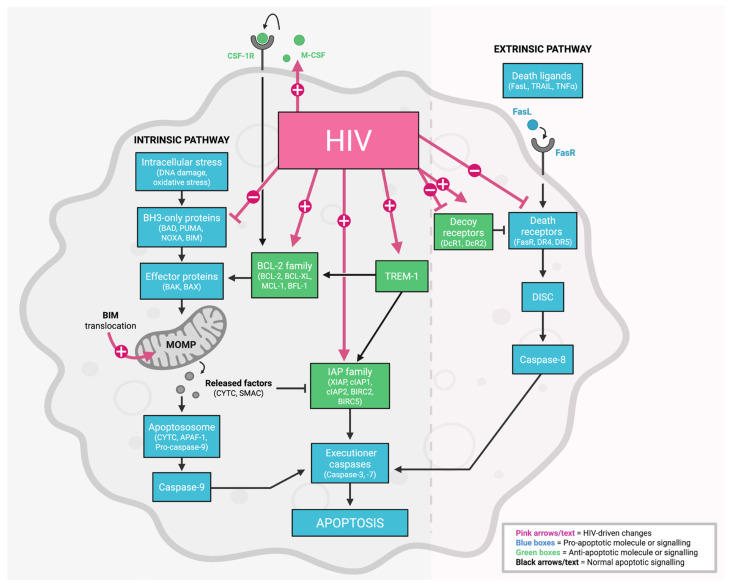
Impact of HIV on the intrinsic and extrinsic apoptosis pathways in macrophages. The intrinsic apoptotic pathway regulates mitochondrial integrity and is initiated by intracellular stress signals, including DNA damage, oxidative stress, growth factor deprivation, and oncogene activation, which converge on activation of BH3-only pro-apoptotic proteins (BAD, PUMA, NOXA, and BIM). BH3-only proteins promote apoptosis by neutralising anti-apoptotic BCL-2 family members and/or directly activating the effector proteins BAX and BAK. Activated BAX and BAK oligomerise at the mitochondrial outer membrane, resulting in mitochondrial outer membrane permeabilization (MOMP), loss of mitochondrial membrane potential, and release of pro-apoptotic factors including cytochrome c and second mitochondria-derived activator of caspases (SMAC) from the intermembrane space. Cytochrome c (CYTC) drives apoptosome assembly through APAF-1 and caspase-9 activation, culminating in activation of executioner caspases-3 and -7 and apoptotic cell death. The extrinsic apoptotic pathway is initiated by engagement of death ligands, including FasL, TRAIL, and TNFα, with their cognate death receptors such as FasR and TRAIL receptors DR4 and DR5. Ligand binding induces receptor trimerization and recruitment of adaptor proteins to form the death-inducing signalling complex (DISC), resulting in caspase-8 activation and subsequent activation of executioner caspases-3 and -7. Extrinsic signalling is further modulated by expression of decoy receptors (DcR1, DcR2) and DISC regulators, which can attenuate caspase-8 activation. Sites at which HIV infection has been reported to modulate apoptotic signalling in macrophages are highlighted in pink within the pathways. Abbreviations: CSF-1R: colony-stimulating factor receptor; M-CSF: macrophage colony-stimulating factor; TNFα: tumour necrosis factor alpha. Created in BioRender. Rikard-Bell, L. (2026) https://BioRender.com/i9s0bf6.

Mechanistic studies primarily implicate the intrinsic apoptosis pathway as a target of HIV-inhibition of macrophage apoptosis, specifically factors within the BCL-2 family [64,68,73,77,79,80,85], although evidence also supports the involvement of the extrinsic pathway (e.g., FasL, TRAIL, tumour necrosis factor alpha (TNFα)) [68,74] (Figure 1).

Intrinsic Pathway. There is consistent and substantial evidence that HIV reprograms the mitochondrial (intrinsic) apoptotic machinery in macrophages (Table 1). Based on current in vitro and ex vivo data, this occurs most commonly through the upregulation of anti-apoptotic BCL-2 family members (BCL-2, BCL-XL, MCL-1) [68,73,77,78,79] and inhibitor of apoptosis (IAP) proteins (cIAP1, cIAP2, XIAP) [68,86]. Collectively, these data support a HIV-driven mitochondrial protection phenotype that promotes macrophage survival [64,68,75,76,77,78,79].

While anti-apoptotic BCL-2/IAP-centred survival signalling represents the most consistent findings, mitochondrial localisation of the pro-apoptotic BIM protein is also observed [77,82] even without accompanying HIV-mediated cytopathogenesis [77]. This suggests that pro-apoptotic signalling or mitochondrial priming could be engaged, but that progression to mitochondrial depolarisation is constrained, potentially by increased anti-apoptotic buffering by BCL-2 or IAP proteins.

Triggering Receptor Expressed on Myeloid Cells 1 (TREM-1) is a member of the immunoglobulin superfamily expressed on neutrophils, monocytes and macrophages that amplifies inflammatory responses to pattern recognition receptor signalling [87]. Several studies have shown upregulated expression of TREM-1 in primary HIV-infected MDMs [77,80,84] and Kupffer cells [84] compared to uninfected cells. In mechanistic studies, HIV proteins Tat and Gp120 were shown to inhibit apoptosis in primary MDMs and macrophage cell lines via TREM-1 signalling [80], linking TREM-1 expression to changes in BCL-2 and caspase activity, reinforcing survival. Additionally, as TREM-1 amplifies pattern recognition receptor signalling, viral activation of these receptors in the liver may also contribute to TREM-1 associated inflammatory responses in HIV infection [84]. Given its role in amplifying pattern recognition receptor signalling, TREM-1 represents a potential therapeutic target to both sensitise HIV-infected macrophages to apoptosis and reduce virus-induced inflammation and comorbidities.

Extrinsic Pathway. Across studies in Table 1, the impact of HIV on death-receptor signalling in macrophages is heterogeneous. Findings diverge regarding macrophage sensitivity to TRAIL-induced apoptosis, reflecting variable expression of death receptors (DR4/DR5) that induce apoptotic signalling versus decoy receptors (DcR1/DcR2) which inhibit signalling by binding to TRAIL, across primary MDMs and macrophage cell lines [68,74]. Macrophage colony-stimulating factor (M-CSF) is elevated in HIV-infected primary MDMs and microglia compared to their uninfected counterparts [68,88]. M-CSF is implicated in dampening TRAIL-mediated apoptosis, with evidence of Env-dependent reductions in DR4/DR5 death receptors, increased anti-apoptotic mediators (e.g., BFL-1, MCL-1), and elevated M-CSF in infected MDMs [68]. Overall, while HIV does modulate extrinsic death-receptor pathways, the evidence is less abundant, more model-dependent, and less consistent than evidence implicating mitochondrial (intrinsic) alterations [77,78,79].

#### 3.2.3. Other (Virus-Mediated) Mechanisms of Macrophage Survival

Beyond apoptosis resistance, macrophage survival during HIV infection can be influenced by other mechanisms such as autophagy and cell cycle regulation. Autophagy can be modulated by HIV proteins to favour viral persistence (reviewed in [89]), but its role in survival of HIV-infected macrophages is less clear. Autophagy may play a role in inhibiting Vpr-induced apoptosis in macrophages [90], while the ability of second mitochondria-derived activator of caspases (SMAC) mimetics to increase apoptosis of HIV-infected macrophages involves enhancing the early stages of autophagy [86]. Further work is required to fully elucidate the interaction of autophagy and apoptosis pathways in HIV-infected macrophages to determine the potential for modulating these pathways to enhance infected cell elimination.

While many macrophage types are considered to be non-dividing, cell cycle pathways also regulate important stress and survival responses [91]. HIV Vpr induces cell cycle arrest in HIV-infected macrophages to enhance replication (reviewed in [92]). While HIV-induced cell cycle arrest has been associated with apoptosis in T cells (reviewed in [93]), whether this occurs also in macrophages in unclear.

The anti-apoptotic long noncoding RNA SAF is highly upregulated in MDMs infected with pseudotyped HIV, but not bystander cells [81]. SAF downregulation increases capase-3/7 protein levels and cell death of HIV+ macrophages, suggesting a role for this long noncoding RNA in conferring apoptosis resistance. Within MDMs, HIV induces degradation of lincRNA-p21, which is involved in mediating apoptosis in response to double stranded DNA breaks, thereby evading apoptosis that may be triggered by HIV integration [94]. Furthermore, this apoptosis-evading mechanism may be unique to macrophages, highlighting key cell-type differences in HIV reservoir cells regarding cell survival mechanisms.

#### 3.2.4. Limitations and Methodological Considerations

While in vitro studies have substantially advanced our understanding of HIV infection dynamics and cell survival in macrophages, several methodological and translational limitations exist which complicate the ability to draw definitive conclusions from multiple studies.

Varying and Non-Physiological Virus and Cell systems. Relatively few studies investigating apoptosis pathways in macrophages explicitly demonstrated that HIV protects macrophages from death using validated experimental indicators of apoptosis. Furthermore, some studies rely on immortalised cell lines, such as HMC3 or THP-1, which are transcriptionally different to primary cells in vitro [95,96] and may not reflect long-lived macrophage reservoirs in vivo. Although chronically HIV-infected myeloid cell lines, including U1 and OM10.1 [97], alongside adapted THP-1 cell lines [28,98], are highly useful models for studying HIV latency in myeloid cells, these models may have similar limitations. Additionally, viral gene expression has been shown to differ significantly between cell lines and ex vivo cells from PWH [99]. Viral strain selection may further complicate interpretation, as T-cell-adapted strains such as LAI and macrophage-tropic strains such as BaL engage distinct entry receptors, differ in their capacity to infect macrophages, and may have different replication kinetics and host–viral interactions [100,101]. Furthermore, laboratory HIV strains may not accurately reflect the behaviour of tissue-resident quasi-species found in compartments such as the central nervous system (CNS) and GALT. Consequently, further studies employing diverse clinical isolates are required to specifically identify altered apoptosis pathways in HIV-infected primary macrophages to more accurately recapitulate the dynamics of the viral reservoir.

Microenvironmental Influence and Macrophage Heterogeneity. Current in vitro models often fail to capture the diversity of tissue-resident macrophages. There is substantial macrophage heterogeneity not only between but also within various anatomical compartments [102,103,104,105] which may have implications for HIV reservoirs and how they can be eliminated [92]. Unlike MDMs, tissue-resident macrophages are long-lived and self-renewing [106], properties that may confer intrinsic differences in apoptotic thresholds and survival programmes. Cytokine-driven polarisation further modulates apoptotic resistance, with M-CSF promoting upregulation of anti-apoptotic mediators including MCL-1, BFL-1, and BCL-2, while granulocyte-macrophage colony-stimulating factor (GM-CSF) downregulates TRAIL death receptors [68]. Together, these observations suggest that sensitivity to apoptosis modulation is likely phenotype- and tissue-dependent. However, to date, relative apoptosis resistance between macrophages derived from different tissues, or between distinct macrophage subtypes within the same tissue, has not been explored. A further limitation to these studies is the investigation of productively-infected cells. Virtually no studies have specifically investigated the apoptotic resistance phenotype of reactivated latently infected myeloid reservoirs.

Confounding Factors in In Vitro Culture. The overwhelming majority of studies employ bulk analysis of HIV-infected macrophage cultures that includes both uninfected bystander and infected cells. This likely masks the resistance phenotype of the persistently infected cells, since studies in CD4+ T cells have demonstrated distinct patterns of cell death in infected versus bystander cells [107]. Differences in culture conditions, such as the use of human versus bovine serum, have been implicated in altering the phenotype and surface receptor expression of MDMs [108]. This variability could contribute to discrepancies in apoptotic signalling, particularly for the extrinsic pathway, potentially explaining conflicting results regarding death receptor expression [68,74].

Methodological Challenges in Apoptosis Measurement. A major limitation of studies to date is the disconnect between mechanistic analyses and concurrent apoptosis measurements. Many studies rely heavily on techniques such as proteomics or gene expression profiling without confirming that the observed molecular changes result in measurable resistance to cell death, underscoring the need for integrated functional assays. Additionally, because apoptotic factors undergo extensive post-translational modifications, including phosphorylation of pro-survival enzymes or the cleavage of pro-caspases, mRNA levels may not accurately reflect the abundance or activation state of the proteins that ultimately execute the apoptotic programme [109].

Detachment of macrophages required for flow cytometric analysis can mechanically cleave surface receptors such as FasR [110] and potentially modify receptor-signalling pathways and impact background cell death [111]. Therefore, microscopy or other assays not requiring cell detachment for analysis of macrophage cell death are preferable. Finally, the choice of apoptosis marker is critical; since different indices evaluate different stages of apoptosis, with some being earlier and potentially more transitory (e.g., Annexin-V) while others reflect a later and more irreversible stage (e.g., TUNEL assay or caspase activity). Using multiple complementary assays is recommended to definitively distinguish apoptosis from other forms of programmed cell death [112].

Moving forward, robust, physiologically relevant in vitro models that employ integrated functional assays coupled with specific analysis of HIV-infected as compared to bystander cells are essential for accurately studying apoptosis and evaluating cure strategies targeting the macrophage HIV reservoir.

## 4. Translational Strategies for Macrophage Reservoir Elimination

Given HIV-infected macrophages exhibit heightened resistance to multiple apoptotic stimuli, targeting this resistance may represent a promising strategy for their elimination. Indeed, a number of approaches have been investigated, with some promising strategies involving small molecule inhibitors like SMAC mimetics, modulating the phosphoinositide-3-kinase (PI3K)/protein kinase B (AKT) pathway as well as combination strategies. These avenues for inducing apoptosis in HIV-infected macrophages are summarised in Table 2.

### 4.1. Small Molecule Inhibitors

AKT inhibitors. HIV infection of macrophages constitutively activates the PI3K/AKT cell survival pathway [113] and AKT inhibition using perifosine promotes cell death and restricts viral production in HIV-infected MDMs and microglial cell lines [114]. The efficacy of more contemporary AKT inhibitors such as capivasertib to modulate death of HIV-infected macrophages remains to be evaluated.

IAP inhibitors/SMAC mimetics. SMAC mimetics are small molecules that mimic endogenous SMAC proteins and trigger the degradation of IAPs, which normally sequester caspases to prevent apoptosis. These agents are also among the most commonly used compounds for targeting HIV-infected macrophages in vitro. SMAC mimetics LCL161 and birinapant have demonstrated efficacy in vitro in inducing selective apoptosis of HIV-infected MDM from uninfected controls [86] and PWH, regardless of ART status [83]. Beyond their pro-apoptotic role, these compounds have shown dual potential as latency reversing agents in CD4+ T cells [121]. However, dose-limiting toxicity issues including adverse neurological events and cytokine release syndrome may limit the clinical use of many SMAC mimetics [122,123,124,125].

CSF-1R antagonists. HIV-1 infection induces production of the pro-survival cytokine M-CSF (CSF-1), which drives resistance to apoptotic stimuli [68]. Consequently, inhibiting the M-CSF receptor (CSF-1R) presents a potential strategy for macrophage elimination. Imatinib and high-affinity CSF-1R inhibitors such as PLX647, PLX3397, and PLX5622, have shown efficacy in selectively killing HIV-infected macrophages by removing these critical survival signals [68,119].

TREM-1 blockade. Given that TREM-1 is upregulated in HIV-infected macrophages (Section 3.2.2) and contributes to an anti-apoptotic phenotype, blocking this receptor or its downstream signalling is an emerging avenue for inducing selective apoptosis [80]. Campbell et al. demonstrated that TREM-1 silencing in HIV-infected macrophages leads to decreased anti-apoptotic proteins and increased cell death, specifically through disruption of mitochondrial membrane potential [77]. TREM-1 silencing increases caspase 3 activation and reduces BCL-2 expression, making macrophages more susceptible to apoptosis [80]. Importantly, silencing of TREM-1 causes HIV-infected microglia to undergo cell death without triggering broader inflammatory responses [78].

### 4.2. Combination Strategies

The failure of initial “shock and kill” strategies to eliminate HIV-infected cells despite efficient viral reactivation has prompted the development of ‘prime, shock, and kill’ approaches which aim to sensitise (“prime”) latently infected cells to cell death by targeting intrinsic survival pathways [126,127]. This strategy has been successfully modelled in CD4+ T cells with the BCL-2 inhibitor Venetoclax which, when combined with latency reversing agents, led to the selective clearance of HIV-infected CD4+ T cells in vitro and ex vivo [126]. Further synergy has been observed when targeting multiple BCL-2 family members simultaneously, such as combining Venetoclax with the MCL-1 inhibitor S63845 in a mouse model of HIV [128]. While the efficacy of these models has not been tested in macrophages, they hold significant potential provided toxicity issues observed in mouse models can be overcome [129].

A similar approach combining latency reversal with inhibition of autophagy and agents to enhance apoptosis has demonstrated efficacy in inducing death of HIV-infected cells both in vivo in humanised mouse models and ex vivo in peripheral blood mononuclear cells (PBMCs) from PWH [130]. This approach was shown to reduce HIV reservoirs in CD4+ T cells, CD11b+ macrophages in the brain, and PBMCs harbouring intact provirus in humanised mice [131]. Chen et al. further confirmed the ability of this approach to clear HIV-infected PBMCs from both ART-experienced and naïve rhesus macaques ex vivo [132], establishing combination prime, shock and kill approaches as promising strategies for potential HIV eradication. However, these approaches have not yet been specifically assessed in human macrophage reservoirs, which will be important to determine their translational relevance for comprehensive HIV reservoir eradication.

### 4.3. Delivery Challenges

The clinical translation of these strategies is hindered by the anatomical sequestration of macrophages in sanctuary sites such as the CNS and GALT [12,17,133,134]. Penetrating the blood–brain barrier remains a major hurdle, necessitating the development of specialised delivery platforms such as nanoparticles. Recent advances have seen nanoparticle technologies adapted for the targeted delivery of ART, including to macrophages [135], and have demonstrated significantly enhanced accumulation within lymph node resident populations [136]. The delivery of mRNA via lipid nanoparticles to reverse latency in HIV-infected CD4+ T cells in vitro has been recently shown to be highly potent with minimal toxicity [137]. Additionally, SMAC-loaded, T-cell membrane-coated nanoparticles selectively killed HIV-infected macrophages via autophagy-dependent apoptosis without off-target effects on bystander cells [116]. This strategy could also enhance the targeting of other drugs that may show promise at eliminating primary macrophages in vitro. Leveraging these platforms to deliver pro-apoptotic priming agents could fundamentally transform HIV cure strategies by ensuring these compounds reach the deep-tissue reservoirs where macrophages persist. However, the pre-clinical validation of these novel strategies is constrained by the limited availability of high-fidelity human macrophage models and standardised measurement assays.

## 5. Limitations in Discovery and Pre-Clinical Targeting of Macrophage Reservoirs

**Figure 2:**
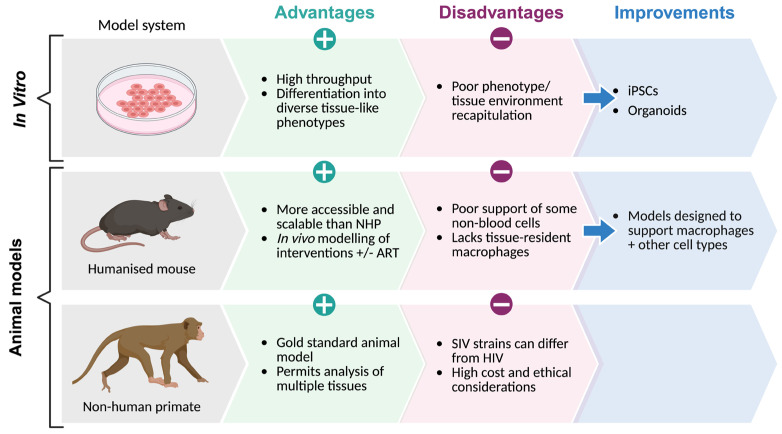
Pre-clinical models for studying HIV-infected macrophage reservoirs. Overview of the strengths and limitations of in vitro and animal models used for studying HIV-infected macrophages, along with potential adaptations to aimed to enhance physiological relevance. These models include cell culture systems, such as cytokine-directed differentiation of monocytes into tissue-like phenotypes [145,146,147], induced pluripotent stem cells (iPSCs), and organoids, as well as humanised mouse models [23,128,148,149] and non-human primate models (NHP) [150]. Created in BioRender. Rikard-Bell, L. (2026) https://BioRender.com/y57b5xn.

The study of macrophage reservoirs remains a major challenge for HIV cure research due to the difficulty of isolating rare, tissue-resident HIV+ macrophages from PWH [2]. Consequently, most mechanistic and therapeutic studies rely on in vitro, ex vivo, and animal models, each with its own distinct advantages and limitations, which are summarised in Figure 2 below. Important advancements to improve pre-clinical investigations of HIV macrophage reservoirs include the use of induced pluripotent stem cell (iPSC)-derived models [138,139,140] and more complex organoid systems, which offer three-dimensional, tissue-like microenvironments and have recently been developed to model HIV infection in the brain [141,142,143,144].

## 6. Clinical Evaluation of Cure Interventions Within Myeloid Reservoirs

Cure interventions have historically prioritised analysis of peripheral blood CD4+ T-cell reservoirs, often overlooking monocyte and tissue-resident macrophage reservoirs. Given sustained, ART-free remission is likely to require comprehensive targeting of diverse anatomical sites and cell types [151], incorporating analysis of myeloid populations in tissue-based sampling will be important to achieve this goal.

**Figure 3:**
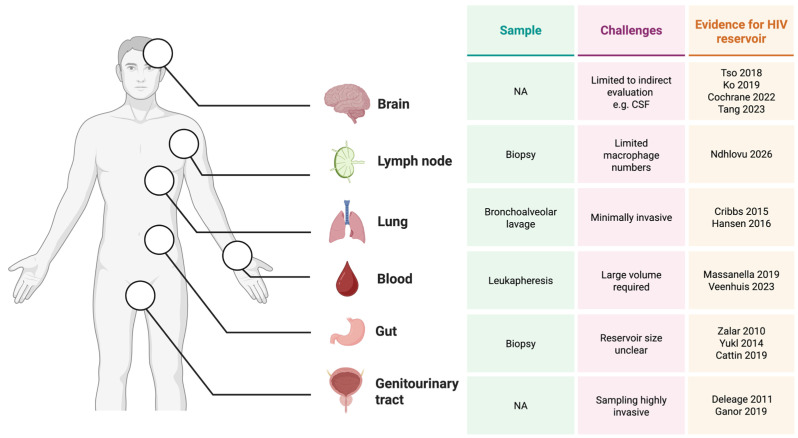
Sampling primary macrophages from PWH on ART. Overview of potential sampling options for key anatomical compartments in which macrophage HIV persistence has been documented, including the brain (via cerebrospinal fluid surrogates), lymph nodes, lungs (via bronchoalveolar lavage), peripheral blood (via leukapheresis), gut, and the male genital tract (urethra) and key technical challenges [6,8,11,12,13,14,15,16,17,19,20,21,133,156]. Created in BioRender. Rikard-Bell, L. (2026) https://BioRender.com/0ija4r0.

A limitation in current cure research is the difficulty in characterising the source of rebound virus, specifically their anatomical origins and cellular lineages, however the latter is possible and has been used to demonstrate the macrophage reservoirs can contribute to viral rebound upon ART interruption [22]. While profiling the cellular reservoir prior to intervention has been applied to T cells [152], this precedent has not yet been extended to myeloid cells and is challenged by low infection frequency and poor yields from tissue biopsies. Despite compelling arguments for including myeloid reservoirs as primary or secondary trial endpoints [153], current protocols rarely incorporate such sampling. Furthermore, observations in peripheral blood often fail to capture the effects of interventions within deep-tissue sanctuaries [154], where viral compartmentalisation and restricted drug penetration may facilitate persistence [155]. Robust evaluation requires pre- and post-intervention measurements of myeloid reservoir size, distribution, and functionality using macrophage-specific assays. The following section briefly discusses tissue sites that represent key priorities for quantifying the macrophage reservoir in clinical trials (Figure 3).

### 6.1. Potential Tissue Site Sampling for Myeloid Reservoir Studies

Blood. Peripheral blood sampling offers accessible means to interrogate the monocyte reservoir but necessitates large-volume leukapheresis to achieve adequate sensitivity. Utilisation of myeloid-adapted versions of the intact proviral DNA assay and quantitative viral outgrowth assay provide a robust and compartment-specific measurement [8] and demonstrate the feasibility of evaluating the monocyte reservoir pre-and post-intervention provided sufficient blood sampling is available. This approach possibly represents the least invasive and most feasible approach for assessing myeloid reservoirs in HIV cure studies.

Gut. While routine gut biopsies, primarily obtained from the colon, enable sampling of tissue-resident immune cells in the GALT, there is limited evidence supporting the existence of a HIV reservoir in GALT macrophages [157]. Despite this, advancements in lamina propria leukocyte isolation, combined with sensitive techniques such as droplet digital polymerase chain reaction or in situ hybridisation, permit high-resolution detection of HIV nucleic acids in gut tissues [158,159,160] and may advance our understanding of myeloid reservoirs in the gut. Importantly, patient acceptability of gut tissue sampling is high when paired with routine cancer screenings [161]. While gut sampling has been incorporated in a number of HIV cure studies, these investigations have not systematically interrogated macrophage populations [162,163,164].

Lung. Bronchoalveolar lavage provides an accessible and high-yielding source of primary tissue macrophages, typically recovering a population of >90% alveolar macrophages [13,26,165]. Given that these macrophages harbour replication-competent HIV during ART [13,18], the lung is a critical and clinically feasible compartment for studying the myeloid reservoir [166].

Other sites. Lymph node sampling has been used to characterise HIV T-cell reservoirs in PWH [167]. Recently, HIV proviral DNA and inducible, multiply spliced RNA has been detected in germinal centre macrophages within the lymph node of PWH with sustained viral suppression [156]. Lymph node biopsies and fine needle aspirates are feasible and well-tolerated [168] but often yield limited macrophage numbers [169]. Similarly, while hepatic macrophages (Kupffer cells) constitute a potential reservoir, there is currently no evidence for replication-competent virus in the liver [170]. Direct assessment of the brain HIV reservoir is not feasible in living participants; consequently, cerebrospinal fluid is often used as a surrogate for CNS viral activity [171] but cannot be attributed to HIV cell type. Substantially more work is required to evaluate whether myeloid HIV reservoirs can be detected or reliably quantified in different tissue samples to determine the clinical utility of including these biopsies in interventional studies, given the invasive nature and associated risk of tissue sampling.

### 6.2. Advanced Analytical Frameworks and Success Criteria

Emerging multi-omic approaches are transforming the capacity to define the macrophage HIV reservoir. In situ hybridisation approaches allow HIV reservoir characterisation within the native tissue environment [172] while spatial transcriptomics (reviewed in [173,174]) offers the potential for high-resolution mapping of the viral microenvironment, including cell-to-cell interactions and viral integration sites [175], although this approach is yet to be fully applied to the analysis of HIV reservoirs. Single-cell RNA sequencing approaches enable cell-specific identification of HIV reservoirs. Recent advancements significantly improve the sensitivity for detecting HIV transcripts and can be combined with surface phenotyping for unparalleled characterisation of the HIV reservoir [176]. When combined with epigenetic profiling, cell phenotyping, viral sequencing, and integration site analysis, these single-cell approaches provide critical insight into reservoir characteristics including clonality and transcriptional competence [177,178,179]. In parallel, the identification of surrogate biomarkers—such as macrophage-specific inflammatory signatures in the brain or lung—could offer indirect but reliable measures of reservoir dynamics and the in vivo efficacy of cure interventions.

## 7. Conclusions and Future Perspectives

The resistance of HIV-infected macrophages to both virus- and immune-mediated elimination mechanisms highlight the critical need to understand cellular and molecular determinants that confer resistance to death. This understanding is essential for developing effective therapeutic strategies to target this persistent reservoir. HIV-infected macrophages exhibit resistance to apoptosis through both intrinsic and extrinsic pathways; thus, therapeutic targeting of these survival mechanisms represents a promising strategy for their elimination and for advancing the HIV cure agenda. Achieving this will require a shift toward tissue-resident macrophage models, such as induced pluripotent stem cell derived systems and organoids, to accurately capture the impact of the tissue microenvironment and macrophage heterogeneity on viral persistence and develop interventions able to penetrate tissue sanctuary sites.

Finally, the implementation of robust myeloid reservoir sampling and measurements before and after therapeutic intervention will be essential for targeting all cellular reservoirs of HIV and achieving sustained HIV remission and thus improving long-term health outcomes for PWH.

## Figures and Tables

**Table 2:** Strategies for eliminating HIV-infected macrophages through the modulation of apoptosis pathways.

HIV Exposure (Tropism)	Cell Type	Treatment	Impact on Viability ^1^	Ref.
***PI3K/AKT pathway inhibitors***				
YU-2 (R5)	MDM	AKT inhibitor IV, AKT inhibitor VIII	↑ SNP-induced apoptosis	Chugh 2008 [113]
BaL (R5)	MDM	Perifosine	↑ SNP-induced cell death	Lucas 2010 [114]
Tat	Microglial cell line (CHME5)	Edelfosine, miltefosine, perifosine	↑ LPS/CHX-induced cell death	Lucas 2010 [114]
Tat	Microglial cell line (CHME5)	Lancemaside A1, arctigenin, Compound K	↑ LPS/CHX-induced cell death	Kim 2011 [115]
***SMAC mimetics***				
BaL (R5)	MDM	LCL-161, AT-406, birinapant	↑ apoptosis	Campbell 2020 [86]
Chronically HIV-infected macrophage cell line (U1)		LCL161	↑ apoptosis in HIV+ cell line vs. uninfected cell line	Caballero 2021 [83]
CS204 (R5X4)	MDM	LCL161	↑ apoptosis	Caballero 2021 [83]
NL4-3-BaL-HSA (R5)	MDM	AEG40730, LCL161	↑ apoptosis in HIV+ vs. bystander cells	Caballero 2021 [83]
Ex vivo	Human MDM from PWH (*n* = 8)	LCL161	↑ cell death in cells from PWH	Caballero 2021 [83]
Vpr	MDM	AEG40730	↑ apoptosis	Busca 2012 [64]
BaL (R5)	MDM	TNP-coated LCL-161, AT-406	↑ cell death (more effective than free drug)	Campbell 2021 [116]
***Other compounds***				
BaL (R5)	MDM	Trimerized TRAIL	↑ apoptosis	Lum 2001 [117]
ADA (R5), BaL (R5), DJV (R5)	MDM	Recombinant TRAIL	↑ apoptosis	Huang 2006 [118]
SG3 with transmitter founder Env	MDM	Anti-DR5 Ab + recombinant TRAIL	↑ apoptosis	Zhu 2011 [74]
BaL (R5)	MDM	TNFα synthesis inhibitor (RP55778)	↑ apoptosis	Guillemard 2004 [73]
Pseudotyped LAI (X4)	MDM	Tyrosine kinase inhibitor (Imatinib)	↑ apoptosis	Swingler 2007 [68]
Pseudotyped NL4-3-HSA (X4)	MDM	Tyrosine kinase inhibitor (Imatinib)	↑ TRAIL-mediated apoptosis	Swingler 2007 [68]
Pseudotyped ADA (R5) and NL4-3-GFP (X4)	MDM	CSF-1R antagonists (PLX647, PLX3397, PLX5622)	↑ TRAIL-mediated loss of HIV+ cells	Cunyat 2016 [119]
Transmitter founder virus (R5)	Macrophage cell line (THP-1), MDM	Inflammatory agonist (Galectin-3)	↑ apoptosis	Xue 2017 [79]
SIVmac251 (R5)	MDM (macaques)	Inflammatory agonist (Galectin-3)	↑ apoptosis	Xue 2017 [79]
BaL (R5)	MDM	Nanoparticle-coated autophagy-inducing peptides	↑ apoptosis	Zhang 2018 [120]
BaL (R5)	MDM	p53 pathway activator (Nutlin3a)	↑ apoptosis	Barichievy 2018 [94]

Abbreviations: PI3K: phosphoinositide-3-kinase; AKT: protein kinase B; SNP: sodium nitroprusside; TNP: T-cell membrane-coated nanoparticle; Ab: antibody; CSF-1R: Colony-Stimulating Factor 1 Receptor; GFP: green fluorescent protein. A “↑” indicates increase. ^1^ All comparing infected culture conditions to uninfected culture conditions unless stated otherwise.

## Data Availability

No new data were created or analysed in this study.

## Abbreviations

The following abbreviations are used in this manuscript:

AKT	Protein kinase B
ART	Antiretroviral therapy
BAD	BCL-2 associated agonist of cell death
BAK	BCL-2 antagonist/killer
BAX	Bcl-2 associated x apoptosis regulator
BCL-2	B-cell lymphoma 2
BCL-XL	B-cell lymphoma-extra large
BFL-1	BCL-2-related protein A1
BIM	BCL-2 Interacting Mediator of cell death
BIRC2	Baculoviral IAP repeat-containing protein 2
CHX	Cycloheximide
cIAP1	Cellular Inhibitor of Apoptosis Protein 1
cIAP2	Cellular Inhibitor of Apoptosis Protein 2
CNS	Central nervous system
CSF-1R	Macrophage colony-stimulating factor receptor
CTL	Cytotoxic lymphocyte
DISC	Death-inducing signalling complex
Env	Envelope
FasL	Fas ligand
FasR	Fas receptor
GALT	Gut-associated lymphoid tissue
GM-CSF	Granulocyte-macrophage colony-stimulating factor
HIV	Human immunodeficiency virus
IAP	Inhibitor of apoptosis
LPS	Lipopolysaccharide
M-CSF	Macrophage colony-stimulating factor
MCL-1	Myeloid cell leukaemia-1
MDM	Monocyte-derived macrophages
MHC-I	Major histocompatibility complex class I
NK	Natural killer
PBMC	Peripheral blood mononuclear cell
PI3K	Phosphoinositide 3-kinase
PWH	People with HIV
SIV	Simian immunodeficiency virus
SMAC	Second mitochondria-derived activator of caspases
TNFα	Tumour necrosis factor alpha
TRAIL	Tumour necrosis factor-related apoptosis-inducing ligand
TREM-1	Triggering Receptor Expressed on Myeloid Cells-1
VCC	Virus-containing compartment
XIAP	X-linked Inhibitor of Apoptosis Protein

## References

[1] Finzi D., Hermankova M., Pierson T., Carruth L.M., Buck C., Chaisson R.E., Quinn T.C., Chadwick K., Margolick J., Brookmeyer R. (1997). Identification of a Reservoir for HIV-1 in Patients on Highly Active Antiretroviral Therapy. Science.

[2] Sengupta S., Siliciano R.F. (2018). Targeting the Latent Reservoir for HIV-1. Immunity.

[3] Siliciano J.D., Siliciano R.F. (2022). In Vivo Dynamics of the Latent Reservoir for HIV-1: New Insights and Implications for Cure. Annu. Rev. Pathol. Mech. Dis..

[4] Crowe S.M., Sonza S. (2000). HIV-1 Can Be Recovered from a Variety of Cells Including Peripheral Blood Monocytes of Patients Receiving Highly Active Antiretroviral Therapy: A Further Obstacle to Eradication. J. Leukoc. Biol..

[5] Ellery P.J., Tippett E., Chiu Y.-L., Paukovics G., Cameron P.U., Solomon A., Lewin S.R., Gorry P.R., Jaworowski A., Greene W.C. (2007). The CD16+ Monocyte Subset Is More Permissive to Infection and Preferentially Harbors HIV-1 In Vivo. J. Immunol..

[6] Massanella M., Bakeman W., Sithinamsuwan P., Fletcher J.L.K., Chomchey N., Tipsuk S., Chalermchai T., Routy J.-P., Ananworanich J., Valcour V.G. (2019). Infrequent HIV Infection of Circulating Monocytes during Antiretroviral Therapy. J. Virol..

[7] Shiramizu B., Gartner S., Williams A., Shikuma C., Ratto-Kim S., Watters M., Aguon J., Valcour V. (2005). Circulating Proviral HIV DNA and HIV-Associated Dementia. AIDS.

[8] Veenhuis R.T., Abreu C.M., Costa P.A.G., Ferreira E.A., Ratliff J., Pohlenz L., Shirk E.N., Rubin L.H., Blankson J.N., Gama L. (2023). Monocyte-Derived Macrophages Contain Persistent Latent HIV Reservoirs. Nat. Microbiol..

[9] Wong M.E., Jaworowski A., Hearps A.C. (2019). The HIV Reservoir in Monocytes and Macrophages. Front. Immunol..

[10] Veenhuis R.T., Abreu C.M., Shirk E.N., Gama L., Clements J.E. (2021). HIV Replication and Latency in Monocytes and Macrophages. Semin. Immunol..

[11] Deleage C., Moreau M., Rioux-Leclercq N., Ruffault A., Jegou B., Dejucq-Rainsford N. (2011). Human Immunodeficiency Virus Infects Human Seminal Vesicles in Vitro and in Vivo. Am. J. Pathol..

[12] Zalar A., Figueroa M.I., Ruibal-Ares B., Bare P., Cahn P., de Bracco M.M., Belmonte L. (2010). Macrophage HIV-1 Infection in Duodenal Tissue of Patients on Long Term HAART. Antivir. Res..

[13] Cribbs S.K., Lennox J., Caliendo A.M., Brown L.A., Guidot D.M. (2015). Healthy HIV-1-Infected Individuals on Highly Active Antiretroviral Therapy Harbor HIV-1 in Their Alveolar Macrophages. AIDS Res. Hum. Retroviruses.

[14] Ko A., Kang G., Hattler J.B., Galadima H.I., Zhang J., Li Q., Kim W.-K. (2019). Macrophages but Not Astrocytes Harbor HIV DNA in the Brains of HIV-1-Infected Aviremic Individuals on Suppressive Antiretroviral Therapy. J. Neuroimmune Pharmacol..

[15] Tso F.Y., Kang G., Kwon E.H., Julius P., Li Q., West J.T., Wood C. (2018). Brain Is a Potential Sanctuary for Subtype C HIV-1 Irrespective of ART Treatment Outcome. PLoS ONE.

[16] Ganor Y., Real F., Sennepin A., Dutertre C.-A., Prevedel L., Xu L., Tudor D., Charmeteau B., Couedel-Courteille A., Marion S. (2019). HIV-1 Reservoirs in Urethral Macrophages of Patients under Suppressive Antiretroviral Therapy. Nat. Microbiol..

[17] Tang Y., Chaillon A., Gianella S., Wong L.M., Li D., Simermeyer T.L., Porrachia M., Ignacio C., Woodworth B., Zhong D. (2023). Brain Microglia Serve as a Persistent HIV Reservoir despite Durable Antiretroviral Therapy. J. Clin. Investig..

[18] Cui J., Meshesha M., Churgulia N., Merlo C., Fuchs E., Breakey J., Jones J., Stivers J.T. (2022). Replication-Competent HIV-1 in Human Alveolar Macrophages and Monocytes despite Nucleotide Pools with Elevated dUTP. Retrovirology.

[19] Hansen E.C., Ransom M., Hesselberth J.R., Hosmane N.N., Capoferri A.A., Bruner K.M., Pollack R.A., Zhang H., Drummond M.B., Siliciano J.M. (2016). Diverse Fates of Uracilated HIV-1 DNA during Infection of Myeloid Lineage Cells. eLife.

[20] Yukl S.A., Sinclair E., Somsouk M., Hunt P.W., Epling L., Killian M., Girling V., Li P., Havlir D.V., Deeks S.G. (2014). A Comparison of Methods for Measuring Rectal HIV Levels Suggests That HIV DNA Resides in Cells Other than CD4+ T Cells, Including Myeloid Cells. AIDS.

[21] Cattin A., Wiche Salinas T.R., Gosselin A., Planas D., Shacklett B., Cohen E.A., Ghali M.P., Routy J.-P., Ancuta P. (2019). HIV-1 Is Rarely Detected in Blood and Colon Myeloid Cells during Viral-Suppressive Antiretroviral Therapy. AIDS.

[22] Andrade V.M., Mavian C., Babic D., Cordeiro T., Sharkey M., Barrios L., Brander C., Martinez-Picado J., Dalmau J., Llano A. (2020). A Minor Population of Macrophage-Tropic HIV-1 Variants Is Identified in Recrudescing Viremia Following Analytic Treatment Interruption. Proc. Natl. Acad. Sci. USA.

[23] Honeycutt J.B., Thayer W.O., Baker C.E., Ribeiro R.M., Lada S.M., Cao Y., Cleary R.A., Hudgens M.G., Richman D.D., Garcia J.V. (2017). HIV Persistence in Tissue Macrophages of Humanized Myeloid-Only Mice during Antiretroviral Therapy. Nat. Med..

[24] Schlachetzki J.C., Gianella S., Ouyang Z., Lana A.J., Yang X., O’Brien S., Challacombe J.F., Gaskill P.J., Jordan-Sciutto K.L., Chaillon A. (2024). Gene Expression and Chromatin Conformation of Microglia in Virally Suppressed People with HIV. Life Sci. Alliance.

[25] Byrnes S.J., Jamal Eddine J., Zhou J., Chalmers E., Wanicek E., Osman N., Jenkins T.A., Roche M., Brew B.J., Estes J.D. (2025). Neuroinflammation Associated with Proviral DNA Persists in the Brain of Virally Suppressed People with HIV. Front. Immunol..

[26] Jambo K.C., Banda D.H., Kankwatira A.M., Sukumar N., Allain T.J., Heyderman R.S., Russell D.G., Mwandumba H.C. (2014). Small Alveolar Macrophages Are Infected Preferentially by HIV and Exhibit Impaired Phagocytic Function. Mucosal Immunol..

[27] Blanco A., Coronado R.A., Arun N., Ma K., Dar R.D., Kieffer C. (2024). Monocyte to Macrophage Differentiation and Changes in Cellular Redox Homeostasis Promote Cell Type-Specific HIV Latency Reactivation. Proc. Natl. Acad. Sci. USA.

[28] Kisaka J.K., Rauch D., Griffith M., Kyei G.B. (2024). A Macrophage-Cell Model of HIV Latency Reveals the Unusual Importance of the Bromodomain Axis. Virol. J..

[29] Wong M.E., Johnson C.J., Hearps A.C., Jaworowski A. (2021). Development of a Novel In Vitro Primary Human Monocyte-Derived Macrophage Model To Study Reactivation of HIV-1 Transcription. J. Virol..

[30] Hendricks C.M., Cordeiro T., Gomes A.P., Stevenson M. (2021). The Interplay of HIV-1 and Macrophages in Viral Persistence. Front. Microbiol..

[31] Reu P., Khosravi A., Bernard S., Mold J.E., Salehpour M., Alkass K., Perl S., Tisdale J., Possnert G., Druid H. (2017). The Lifespan and Turnover of Microglia in the Human Brain. Cell Rep..

[32] Gordon S., Plüddemann A. (2017). Tissue Macrophages: Heterogeneity and Functions. BMC Biol..

[33] Wang J., Kubes P. (2016). A Reservoir of Mature Cavity Macrophages That Can Rapidly Invade Visceral Organs to Affect Tissue Repair. Cell.

[34] Shi C., Pamer E.G. (2011). Monocyte Recruitment during Infection and Inflammation. Nat. Rev. Immunol..

[35] Mu X., Li Y., Fan G.-C. (2021). Tissue-Resident Macrophages in the Control of Infection and Resolution of Inflammation. Shock.

[36] Silvin A., Qian J., Ginhoux F. (2023). Brain Macrophage Development, Diversity and Dysregulation in Health and Disease. Cell. Mol. Immunol..

[37] Hou F., Xiao K., Tang L., Xie L. (2021). Diversity of Macrophages in Lung Homeostasis and Diseases. Front. Immunol..

[38] Tan S.Y.S., Krasnow M.A. (2016). Developmental Origin of Lung Macrophage Diversity. Development.

[39] Gu Y., Lawrence T., Mohamed R., Liang Y., Yahaya B.H. (2022). The Emerging Roles of Interstitial Macrophages in Pulmonary Fibrosis: A Perspective from scRNA-Seq Analyses. Front. Immunol..

[40] Lin Z., Zheng Y., Zhong Y., Wang H. (2026). Single-Cell Insights Into Macrophage Subtypes in Pulmonary Infections. Adv. Sci..

[41] Zhou D., Huang C., Lin Z., Zhan S., Kong L., Fang C., Li J. (2014). Macrophage Polarization and Function with Emphasis on the Evolving Roles of Coordinated Regulation of Cellular Signaling Pathways. Cell. Signal..

[42] Ferreira E.A., Clements J.E., Veenhuis R.T. (2024). HIV-1 Myeloid Reservoirs—Contributors to Viral Persistence and Pathogenesis. Curr. HIV/AIDS Rep..

[43] Calantone N., Wu F., Klase Z., Deleage C., Perkins M., Matsuda K., Thompson E.A., Ortiz A.M., Vinton C.L., Ourmanov I. (2014). Tissue Myeloid Cells in SIV-Infected Primates Acquire Viral DNA through Phagocytosis of Infected T Cells. Immunity.

[44] Baxter A.E., Russell R.A., Duncan C.J., Moore M.D., Willberg C.B., Pablos J.L., Finzi A., Kaufmann D.E., Ochsenbauer C., Kappes J.C. (2014). Macrophage Infection via Selective Capture of HIV-1-Infected CD4+ T Cells. Cell Host Microbe.

[45] Kruize Z., Kootstra N.A. (2019). The Role of Macrophages in HIV-1 Persistence and Pathogenesis. Front. Microbiol..

[46] Zou Z., Chastain A., Moir S., Ford J., Trandem K., Martinelli E., Cicala C., Crocker P., Arthos J., Sun P.D. (2011). Siglecs Facilitate HIV-1 Infection of Macrophages through Adhesion with Viral Sialic Acids. PLoS ONE.

[47] Gaudin R., Berre S., Cunha De Alencar B., Decalf J., Schindler M., Gobert F.-X., Jouve M., Benaroch P. (2013). Dynamics of HIV-Containing Compartments in Macrophages Reveal Sequestration of Virions and Transient Surface Connections. PLoS ONE.

[48] Koppensteiner H., Banning C., Schneider C., Hohenberg H., Schindler M. (2012). Macrophage Internal HIV-1 Is Protected from Neutralizing Antibodies. J. Virol..

[49] Sharova N., Swingler C., Sharkey M., Stevenson M. (2005). Macrophages Archive HIV-1 Virions for Dissemination in Trans. EMBO J..

[50] Clayton K.L., Collins D.R., Lengieza J., Ghebremichael M., Dotiwala F., Lieberman J., Walker B.D. (2018). Resistance of HIV-Infected Macrophages to CD8(+) T Lymphocyte-Mediated Killing Drives Activation of the Immune System. Nat. Immunol..

[51] Clayton K.L., Mylvaganam G., Villasmil-Ocando A., Stuart H., Maus M.V., Rashidian M., Ploegh H.L., Walker B.D. (2021). HIV-Infected Macrophages Resist Efficient NK Cell-Mediated Killing While Preserving Inflammatory Cytokine Responses. Cell Host Microbe.

[52] Rainho J.N., Martins M.A., Cunyat F., Watkins I.T., Watkins D.I., Stevenson M. (2015). Nef Is Dispensable for Resistance of Simian Immunodeficiency Virus-Infected Macrophages to CD8+ T Cell Killing. J. Virol..

[53] Vojnov L., Martins M.A., Bean A.T., Veloso de Santana M.G., Sacha J.B., Wilson N.A., Bonaldo M.C., Galler R., Stevenson M., Watkins D.I. (2012). The Majority of Freshly Sorted Simian Immunodeficiency Virus (SIV)-Specific CD8(+) T Cells Cannot Suppress Viral Replication in SIV-Infected Macrophages. J. Virol..

[54] Kek H., Laumaea A., Parise S., Poumbourios P., Hearps A.C., Jaworowski A. (2021). Differential Expression of HIV Envelope Epitopes on the Surface of HIV-Infected Macrophages and CD4+ T Cells. Antivir. Res..

[55] Elmore S. (2007). Apoptosis: A Review of Programmed Cell Death. Toxicol. Pathol..

[56] Paul S., Lal G. (2017). The Molecular Mechanism of Natural Killer Cells Function and Its Importance in Cancer Immunotherapy. Front. Immunol..

[57] Brown A., Gartner S., Kawano T., Benoit N., Cheng-Mayer C. (2005). HLA-A2 down-Regulation on Primary Human Macrophages Infected with an M-Tropic EGFP-Tagged HIV-1 Reporter Virus. J. Leukoc. Biol..

[58] Akiyama H., Gummuluru S. (2020). HIV-1 Persistence and Chronic Induction of Innate Immune Responses in Macrophages. Viruses.

[59] Mueller Y.M., De Rosa S.C., Hutton J.A., Witek J., Roederer M., Altman J.D., Katsikis P.D. (2001). Increased CD95/Fas-Induced Apoptosis of HIV-Specific CD8+ T Cells. Immunity.

[60] Doitsh G., Galloway N.L.K., Geng X., Yang Z., Monroe K.M., Zepeda O., Hunt P.W., Hatano H., Sowinski S., Muñoz-Arias I. (2014). Cell Death by Pyroptosis Drives CD4 T-Cell Depletion in HIV-1 Infection. Nature.

[61] Le Douce V., Herbein G., Rohr O., Schwartz C. (2010). Molecular Mechanisms of HIV-1 Persistence in the Monocyte-Macrophage Lineage. Retrovirology.

[62] Kiener P.A., Davis P.M., Starling G.C., Mehlin C., Klebanoff S.J., Ledbetter J.A., Liles W.C. (1997). Differential Induction of Apoptosis by Fas–Fas Ligand Interactions in Human Monocytes and Macrophages. J. Exp. Med..

[63] Perlman H., Pagliari L.J., Georganas C., Mano T., Walsh K., Pope R.M. (1999). Flice-Inhibitory Protein Expression during Macrophage Differentiation Confers Resistance to FAS-Mediated Apoptosis. J. Exp. Med..

[64] Busca A., Saxena M., Kumar A. (2012). Critical Role for Antiapoptotic Bcl-xL and Mcl-1 in Human Macrophage Survival and Cellular IAP1/2 (cIAP1/2) in Resistance to HIV-Vpr-Induced Apoptosis. J. Biol. Chem..

[65] Sonza S., Kiernan R.E., Maerz A.L., Deacon N.J., McPhee D.A., Crowe S.M. (1994). Accumulation of Unintegrated Circular Viral DNA in Monocytes and Growth-Arrested T Cells Following Infection with HIV-1. J. Leukoc. Biol..

[66] Crowe S., Zhu T., Muller W.A. (2003). The Contribution of Monocyte Infection and Trafficking to Viral Persistence, and Maintenance of the Viral Reservoir in HIV Infection. J. Leukoc. Biol..

[67] Aquaro S., Bagnarelli P., Guenci T., De Luca A., Clementi M., Balestra E., Caliò R., Perno C. (2002). Long-term Survival and Virus Production in Human Primary Macrophages Infected by Human Immunodeficiency Virus. J. Med. Virol..

[68] Swingler S., Mann A.M., Zhou J., Swingler C., Stevenson M. (2007). Apoptotic Killing of HIV-1–Infected Macrophages Is Subverted by the Viral Envelope Glycoprotein. PLoS Pathog..

[69] Busca A., Saxena M., Kryworuchko M., Kumar A. (2009). Anti-Apoptotic Genes in the Survival of Monocytic Cells During Infection. Curr. Genom..

[70] Trahtemberg U., Mevorach D. (2017). Apoptotic Cells Induced Signaling for Immune Homeostasis in Macrophages and Dendritic Cells. Front. Immunol..

[71] Laforge M., Campillo-Gimenez L., Monceaux V., Cumont M.-C., Hurtrel B., Corbeil J., Zaunders J., Elbim C., Estaquier J. (2011). HIV/SIV Infection Primes Monocytes and Dendritic Cells for Apoptosis. PLoS Pathog..

[72] Giri M.S., Nebozyhn M., Raymond A., Gekonge B., Hancock A., Creer S., Nicols C., Yousef M., Foulkes A.S., Mounzer K. (2009). Circulating Monocytes in HIV-1-Infected Viremic Subjects Exhibit an Antiapoptosis Gene Signature and Virus- and Host-Mediated Apoptosis Resistance. J. Immunol..

[73] Guillemard E., Jacquemot C., Aillet F., Schmitt N., Barre-Sinoussi F., Israel N. (2004). Human Immunodeficiency Virus 1 Favors the Persistence of Infection by Activating Macrophages through TNF. Virology.

[74] Zhu D.-M., Shi J., Liu S., Liu Y., Zheng D. (2011). HIV Infection Enhances TRAIL-Induced Cell Death in Macrophage by Down-Regulating Decoy Receptor Expression and Generation of Reactive Oxygen Species. PLoS ONE.

[75] Chugh P., Fan S., Planelles V., Maggirwar S.B., Dewhurst S., Kim B. (2007). Infection of Human Immunodeficiency Virus and Intracellular Viral Tat Protein Exert a Pro-Survival Effect in a Human Microglial Cell Line. J. Mol. Biol..

[76] Cosenza M.A., Zhao M.-L., Lee S.C. (2004). HIV-1 Expression Protects Macrophages and Microglia from Apoptotic Death. Neuropathol. Appl. Neurobiol..

[77] Campbell G.R., To R.K., Spector S.A. (2019). TREM-1 Protects HIV-1-Infected Macrophages from Apoptosis through Maintenance of Mitochondrial Function. mBio.

[78] Campbell G.R., Rawat P., To R.K., Spector S.A. (2023). HIV-1 Tat Upregulates TREM1 Expression in Human Microglia. J. Immunol..

[79] Xue J., Fu C., Cong Z., Peng L., Peng Z., Chen T., Wang W., Jiang H., Wei Q., Qin C. (2017). Galectin-3 Promotes Caspase-independent Cell Death of HIV-1-infected Macrophages. FEBS J..

[80] Yuan Z., Fan X., Staitieh B., Bedi C., Spearman P., Guidot D.M., Sadikot R.T. (2017). HIV-Related Proteins Prolong Macrophage Survival through Induction of Triggering Receptor Expressed on Myeloid Cells-1. Sci. Rep..

[81] Boliar S., Gludish D.W., Jambo K.C., Kamng’ona R., Mvaya L., Mwandumba H.C., Russell D.G. (2019). Inhibition of the lncRNA SAF Drives Activation of Apoptotic Effector Caspases in HIV-1–Infected Human Macrophages. Proc. Natl. Acad. Sci. USA.

[82] Castellano P., Prevedel L., Eugenin E.A. (2017). HIV-Infected Macrophages and Microglia That Survive Acute Infection Become Viral Reservoirs by a Mechanism Involving Bim. Sci. Rep..

[83] Caballero R.E., Dong S.X.M., Gajanayaka N., Ali H., Cassol E., Cameron W.D., Korneluk R., Tremblay M.J., Angel J.B., Kumar A. (2021). Role of RIPK1 in SMAC Mimetics-Induced Apoptosis in Primary Human HIV-Infected Macrophages. Sci. Rep..

[84] Hyun J., McMahon R.S., Lang A.L., Edwards J.S., Badilla A.D., Greene M.E., Stone G.W., Pallikkuth S., Stevenson M., Dykxhoorn D.M. (2019). HIV and HCV Augments Inflammatory Responses through Increased TREM-1 Expression and Signaling in Kupffer and Myeloid Cells. PLoS Pathog..

[85] Zhang M., Li X., Pang X., Ding L., Wood O., Clouse K.A., Hewlett I., Dayton A.I. (2002). Bcl-2 Upregulation by HIV-1 Tat during Infection of Primary Human Macrophages in Culture. J. Biomed. Sci..

[86] Campbell G.R., To R.K., Zhang G., Spector S.A. (2020). SMAC Mimetics Induce Autophagy-Dependent Apoptosis of HIV-1-Infected Macrophages. Cell Death Dis..

[87] Roe K., Gibot S., Verma S. (2014). Triggering Receptor Expressed on Myeloid Cells-1 (TREM-1): A New Player in Antiviral Immunity?. Front. Microbiol..

[88] Irons D.L., Meinhardt T., Allers C., Kuroda M.J., Kim W. (2019). Overexpression and Activation of Colony-stimulating Factor 1 Receptor in the SIV/Macaque Model of HIV Infection and neuroHIV. Brain Pathol..

[89] Klute S., Sparrer K.M.J. (2024). Friends and Foes: The Ambivalent Role of Autophagy in HIV-1 Infection. Viruses.

[90] Zhou H.Y., Zheng Y.H., He Y., Chen Z., He B. (2017). The Role of Autophagy in THP-1 Macrophages Resistance to HIV-Vpr-Induced Apoptosis. Exp. Cell Res..

[91] Dillac L., El Dika L., Ullah R., Kubiak J.Z., Kloc M., Kloc M., Kubiak J.Z., Halasa M. (2024). Macrophage Cell Cycle. Monocytes and Macrophages in Development, Regeneration, and Disease.

[92] Ferreira I.A.T.M., Porterfield J.Z., Gupta R.K., Mlcochova P. (2020). Cell Cycle Regulation in Macrophages and Susceptibility to HIV-1. Viruses.

[93] Andersen J.L., Le Rouzic E., Planelles V. (2008). HIV-1 Vpr: Mechanisms of G2 Arrest and Apoptosis. Exp. Mol. Pathol..

[94] Barichievy S., Naidoo J., Boullé M., Scholefield J., Parihar S.P., Coussens A.K., Brombacher F., Sigal A., Mhlanga M.M. (2018). Viral Apoptosis Evasion via the MAPK Pathway by Use of a Host Long Noncoding RNA. Front. Cell. Infect. Microbiol..

[95] Kohro T., Tanaka T., Murakami T., Wada Y., Aburatani H., Hamakubo T., Kodama T. (2004). A Comparison of Differences in the Gene Expression Profiles of Phorbol 12-Myristate 13-Acetate Differentiated THP-1 Cells and Human Monocyte-Derived Macrophage. J. Atheroscler. Thromb..

[96] Rai M.A., Hammonds J., Pujato M., Mayhew C., Roskin K., Spearman P. (2020). Comparative Analysis of Human Microglial Models for Studies of HIV Replication and Pathogenesis. Retrovirology.

[97] Poli G., Poli G., Vicenzi E., Romerio F. (2022). U1 and OM10.1. Myeloid Cell Lines as Surrogate Models of Reversible Proviral Latency. HIV Reservoirs.

[98] Mehla R., Bivalkar-Mehla S., Zhang R., Handy I., Albrecht H., Giri S., Nagarkatti P., Nagarkatti M., Chauhan A. (2010). Bryostatin Modulates Latent HIV-1 Infection via PKC and AMPK Signaling but Inhibits Acute Infection in a Receptor Independent Manner. PLoS ONE.

[99] Telwatte S., Morón-López S., Aran D., Kim P., Hsieh C., Joshi S., Montano M., Greene W.C., Butte A.J., Wong J.K. (2019). Heterogeneity in HIV and Cellular Transcription Profiles in Cell Line Models of Latent and Productive Infection: Implications for HIV Latency. Retrovirology.

[100] Picchio G.R., Gulizia R.J., Wehrly K., Chesebro B., Mosier D.E. (1998). The Cell Tropism of Human Immunodeficiency Virus Type 1 Determines the Kinetics of Plasma Viremia in SCID Mice Reconstituted with Human Peripheral Blood Leukocytes. J. Virol..

[101] Han M., Cantaloube-Ferrieu V., Xie M., Armani-Tourret M., Woottum M., Pagès J.-C., Colin P., Lagane B., Benichou S. (2022). HIV-1 Cell-to-Cell Spread Overcomes the Virus Entry Block of Non-Macrophage-Tropic Strains in Macrophages. PLoS Pathog..

[102] Gerganova G., Riddell A., Miller A.A. (2022). CNS Border-Associated Macrophages in the Homeostatic and Ischaemic Brain. Pharmacol. Ther..

[103] Sankowski R., Böttcher C., Masuda T., Geirsdottir L., Sagar, Sindram E., Seredenina T., Muhs A., Scheiwe C., Shah M.J. (2019). Mapping Microglia States in the Human Brain through the Integration of High-Dimensional Techniques. Nat. Neurosci..

[104] Mould K.J., Moore C.M., McManus S.A., McCubbrey A.L., McClendon J.D., Griesmer C.L., Henson P.M., Janssen W.J. (2021). Airspace Macrophages and Monocytes Exist in Transcriptionally Distinct Subsets in Healthy Adults. Am. J. Respir. Crit. Care Med..

[105] Domanska D., Majid U., Karlsen V.T., Merok M.A., Beitnes A.-C.R., Yaqub S., Bækkevold E.S., Jahnsen F.L. (2022). Single-Cell Transcriptomic Analysis of Human Colonic Macrophages Reveals Niche-Specific Subsets. J. Exp. Med..

[106] Perdiguero E.G., Geissmann F. (2016). The Development and Maintenance of Resident Macrophages. Nat. Immunol..

[107] Cao D., Khanal S., Wang L., Li Z., Zhao J., Nguyen L.N., Nguyen L.N.T., Dang X., Schank M., Thakuri B.K.C. (2021). A Matter of Life or Death: Productively Infected and Bystander CD4 T Cells in Early HIV Infection. Front. Immunol..

[108] Marek N., Wolff A.S.B., Dongre H.N., Suliman S. (2025). Optimizing Monocyte-Derived Immune Cell Cultures: Comparing Xeno-Free and Xenogeneic Conditions. Front. Immunol..

[109] Castellano P., Prevedel L., Valdebenito S., Eugenin E.A. (2019). HIV Infection and Latency Induce a Unique Metabolic Signature in Human Macrophages. Sci. Rep..

[110] Lai T.-Y., Cao J., Ou-Yang P., Tsai C.-Y., Lin C.-W., Chen C.-C., Tsai M.-K., Lee C.-Y. (2022). Different Methods of Detaching Adherent Cells and Their Effects on the Cell Surface Expression of Fas Receptor and Fas Ligand. Sci. Rep..

[111] Feuerer N., Morschl J., Daum R., Weiss M., Hinderer S., Schenke-Layland K., Shipp C. (2021). Macrophage Retrieval from 3D Biomaterials: A Detailed Comparison of Common Dissociation Methods. J. Immunol. Regen. Med..

[112] Kari S., Subramanian K., Altomonte I.A., Murugesan A., Yli-Harja O., Kandhavelu M. (2022). Programmed Cell Death Detection Methods: A Systematic Review and a Categorical Comparison. Apoptosis.

[113] Chugh P., Bradel-Tretheway B., Monteiro-Filho C.M., Planelles V., Maggirwar S.B., Dewhurst S., Kim B. (2008). Akt Inhibitors as an HIV-1 Infected Macrophage-Specific Anti-Viral Therapy. Retrovirology.

[114] Lucas A., Kim Y., Rivera-Pabon O., Chae S., Kim D.H., Kim B. (2010). Targeting the PI3K/Akt Cell Survival Pathway to Induce Cell Death of HIV-1 Infected Macrophages with Alkylphospholipid Compounds. PLoS ONE.

[115] Kim Y., Hollenbaugh J.A., Kim D.H., Kim B. (2011). Novel PI3K/Akt Inhibitors Screened by the Cytoprotective Function of Human Immunodeficiency Virus Type 1 Tat. PLoS ONE.

[116] Campbell G.R., Zhuang J., Zhang G., Landa I., Kubiatowicz L.J., Dehaini D., Fang R.H., Zhang L., Spector S.A. (2021). CD4+ T Cell-Mimicking Nanoparticles Encapsulating DIABLO/SMAC Mimetics Broadly Neutralize HIV-1 and Selectively Kill HIV-1-Infected Cells. Theranostics.

[117] Lum J.J., Pilon A.A., Sanchez-Dardon J., Phenix B.N., Kim J.E., Mihowich J., Jamison K., Hawley-Foss N., Lynch D.H., Badley A.D. (2001). Induction of Cell Death in Human Immunodeficiency Virus-Infected Macrophages and Resting Memory CD4 T Cells by TRAIL/Apo2L. J. Virol..

[118] Huang Y., Erdmann N., Peng H., Herek S., Davis J.S., Luo X., Ikezu T., Zheng J. (2006). TRAIL-Mediated Apoptosis in HIV-1-Infected Macrophages Is Dependent on the Inhibition of Akt-1 Phosphorylation. J. Immunol..

[119] Cunyat F., Rainho J.N., West B., Swainson L., McCune J.M., Stevenson M. (2016). Colony-Stimulating Factor 1 Receptor Antagonists Sensitize Human Immunodeficiency Virus Type 1-Infected Macrophages to TRAIL-Mediated Killing. J. Virol..

[120] Zhang G., Luk B.T., Hamidy M., Zhang L., Spector S.A. (2018). Induction of a Na^+^/K^+^-ATPase-Dependent Form of Autophagy Triggers Preferential Cell Death of Human Immunodeficiency Virus Type-1-Infected Macrophages. Autophagy.

[121] Sampey G.C., Irlbeck D.M., Browne E.P., Kanke M., McAllister A.B., Ferris R.G., Brehm J.H., Favre D., Routy J.-P., Jones C.D. (2018). The SMAC Mimetic AZD5582 Is a Potent HIV Latency Reversing Agent. bioRxiv.

[122] Senzer N.N., LoRusso P., Martin L.P., Schilder R.J., Amaravadi R.K., Papadopoulos K.P., Segota Z.E., Weng D.E., Graham M., Adjei A.A. (2013). Phase II Clinical Activity and Tolerability of the SMAC-Mimetic Birinapant (TL32711) plus Irinotecan in Irinotecan-Relapsed/Refractory Metastatic Colorectal Cancer. J. Clin. Oncol..

[123] Noonan A.M., Bunch K.P., Chen J., Herrmann M.A., Lee J., Kohn E.C., O’Sullivan C.C., Jordan E., Houston N., Takebe N. (2016). Pharmacodynamic Markers and Clinical Results from the Phase 2 Study of the SMAC Mimetic Birinapant in Women with Relapsed Platinum-resistant or -refractory Epithelial Ovarian Cancer. Cancer.

[124] Infante J.R., Dees E.C., Olszanski A.J., Dhuria S.V., Sen S., Cameron S., Cohen R.B. (2014). Phase I Dose-Escalation Study of LCL161, an Oral Inhibitor of Apoptosis Proteins Inhibitor, in Patients With Advanced Solid Tumors. J. Clin. Oncol..

[125] Tolcher A.W., Bendell J.C., Papadopoulos K.P., Burris H.A., Patnaik A., Fairbrother W.J., Wong H., Budha N., Darbonne W.C., Peale F. (2016). A Phase I Dose-Escalation Study Evaluating the Safety Tolerability and Pharmacokinetics of CUDC-427, a Potent, Oral, Monovalent IAP Antagonist, in Patients with Refractory Solid Tumors. Clin. Cancer Res..

[126] Cummins N.W., Sainski A.M., Dai H., Natesampillai S., Pang Y.P., Bren G.D., de Araujo Correia M.C.M., Sampath R., Rizza S.A., O’Brien D. (2016). Prime, Shock, and Kill: Priming CD4 T Cells from HIV Patients with a BCL-2 Antagonist before HIV Reactivation Reduces HIV Reservoir Size. J. Virol..

[127] Chandrasekar A.P., Badley A.D. (2022). Prime, Shock and Kill: BCL-2 Inhibition for HIV Cure. Front. Immunol..

[128] Arandjelovic P., Kim Y., Cooney J.P., Preston S.P., Doerflinger M., McMahon J.H., Garner S.E., Zerbato J.M., Roche M., Tumpach C. (2023). Venetoclax, Alone and in Combination with the BH3 Mimetic S63845, Depletes HIV-1 Latently Infected Cells and Delays Rebound in Humanized Mice. Cell Rep. Med..

[129] Grabow S., Delbridge A.R.D., Aubrey B.J., Vandenberg C.J., Strasser A. (2016). Loss of a Single Mcl-1 Allele Inhibits MYC-Driven Lymphomagenesis by Sensitizing Pro-B Cells to Apoptosis. Cell Rep..

[130] Li M., Liu W., Bauch T., Graviss E.A., Arduino R.C., Kimata J.T., Chen M., Wang J. (2020). Clearance of HIV Infection by Selective Elimination of Host Cells Capable of Producing HIV. Nat. Commun..

[131] Li M., Truong K., Sharma S., Sun B., Chen M., Kimata J.T., Wang J. (2025). Elimination of Human Immunodeficiency Virus Reservoirs Harboring Intact Proviruses. J. Infect. Dis..

[132] Chen M., Li M., Budai M.M., Rice A.P., Kimata J.T., Mohan M., Wang J. (2022). Clearance of HIV-1 or SIV Reservoirs by Promotion of Apoptosis and Inhibition of Autophagy: Targeting Intracellular Molecules in Cure-Directed Strategies. J. Leukoc. Biol..

[133] Cochrane C.R., Angelovich T.A., Byrnes S.J., Waring E., Guanizo A.C., Trollope G.S., Zhou J., Vue J., Senior L., Wanicek E. (2022). Intact HIV Proviruses Persist in the Brain Despite Viral Suppression with ART. Ann. Neurol..

[134] Josefsson L., Von Stockenstrom S., Faria N.R., Sinclair E., Bacchetti P., Killian M., Epling L., Tan A., Ho T., Lemey P. (2013). The HIV-1 Reservoir in Eight Patients on Long-Term Suppressive Antiretroviral Therapy Is Stable with Few Genetic Changes over Time. Proc. Natl. Acad. Sci. USA.

[135] Terkimbi S.D., Dangana R.S., Mbina S.A., Paul-Chima U.O., Aja P.M., Mujinya R. (2026). Nanoparticles in HIV Treatment for Improved Drug Delivery, Clinical Translation, and Future Direction. Discov. Nano.

[136] Fofana J., Zahara S., Chan T., Zang H., Reinhard B.M., Gummuluru S. (2025). Enhancing Antiretroviral Delivery to Secondary Lymph Nodes by Targeting CD169 + Macrophages with HIV-Mimicking Nanoparticles. Sci. Rep..

[137] Cevaal P.M., Kan S., Fisher B.M., Moso M.A., Tan A., Liu H., Ali A., Tanaka K., Shepherd R.A., Kim Y. (2025). Efficient mRNA Delivery to Resting T Cells to Reverse HIV Latency. Nat. Commun..

[138] Eltalkhawy Y.M., Takahashi N., Ariumi Y., Shimizu J., Miyazaki K., Senju S., Suzu S. (2023). iPS Cell-Derived Model to Study the Interaction between Tissue Macrophage and HIV-1. J. Leukoc. Biol..

[139] Takata K., Kozaki T., Lee C.Z.W., Thion M.S., Otsuka M., Lim S., Utami K.H., Fidan K., Park D.S., Malleret B. (2017). Induced-Pluripotent-Stem-Cell-Derived Primitive Macrophages Provide a Platform for Modeling Tissue-Resident Macrophage Differentiation and Function. Immunity.

[140] Wilgenburg B.V., Browne C., Vowles J., Cowley S.A. (2013). Efficient, Long Term Production of Monocyte-Derived Macrophages from Human Pluripotent Stem Cells under Partly-Defined and Fully-Defined Conditions. PLoS ONE.

[141] Selvam S., Dos Reis R.S., Wagner M.C.E., Krishnakumar R., Keshavarz K., Ebrahimkhani M.R., Ayyavoo V. (2026). Vascularized Human Brain Organoids as a Model of the Brain–Peripheral Axis in HIV-1 Neuropathogenesis. Sci. Rep..

[142] Gumbs S.B.H., Berdenis Van Berlekom A., Kübler R., Schipper P.J., Gharu L., Boks M.P., Ormel P.R., Wensing A.M.J., De Witte L.D., Nijhuis M. (2022). Characterization of HIV-1 Infection in Microglia-Containing Human Cerebral Organoids. Viruses.

[143] Donadoni M., Cakir S., Bellizzi A., Swingler M., Sariyer I.K. (2024). Modeling HIV-1 Infection and NeuroHIV in hiPSCs-Derived Cerebral Organoid Cultures. J. Neurovirol..

[144] Narasipura S.D., Zayas J.P., Ash M.K., Reyes A.F., Shull T., Gambut S., Szczerkowski J.L.A., McKee C., Schneider J.R., Lorenzo-Redondo R. (2025). Inflammatory Responses Revealed through HIV Infection of Microglia-Containing Cerebral Organoids. J. Neuroinflamm..

[145] Akagawa K.S. (2002). Functional Heterogeneity of Colony-Stimulating Factor-Induced Human Nonocyte-Derived Macrophages. Int. J. Hematol..

[146] Lescoat A., Ballerie A., Augagneur Y., Morzadec C., Vernhet L., Fardel O., Jégo P., Jouneau S., Lecureur V. (2018). Distinct Properties of Human M-CSF and GM-CSF Monocyte-Derived Macrophages to Simulate Pathological Lung Conditions In Vitro: Application to Systemic and Inflammatory Disorders with Pulmonary Involvement. Int. J. Mol. Sci..

[147] Ryan K.J., White C.C., Patel K., Xu J., Olah M., Replogle J.M., Frangieh M., Cimpean M., Winn P., McHenry A. (2017). A Human Microglia-like Cellular Model for Assessing the Effects of Neurodegenerative Disease Gene Variants. Sci. Transl. Med..

[148] Agarwal Y., Beatty C., Biradar S., Castronova I., Ho S., Melody K., Bility M.T. (2020). Moving beyond the Mousetrap: Current and Emerging Humanized Mouse and Rat Models for Investigating Prevention and Cure Strategies against HIV Infection and Associated Pathologies. Retrovirology.

[149] Zhang C., Zaman L.A., Poluektova L.Y., Gorantla S., Gendelman H.E., Dash P.K. (2023). Humanized Mice for Studies of HIV-1 Persistence and Elimination. Pathogens.

[150] Terrade G., Huot N., Petitdemange C., Lazzerini M., Orta Resendiz A., Jacquelin B., Müller-Trutwin M. (2021). Interests of the Non-Human Primate Models for HIV Cure Research. Vaccines.

[151] Joseph J., Daley W., Lawrence D., Lorenzo E., Perrin P., Rao V.R., Tsai S.Y., Varthakavi V. (2022). Role of Macrophages in HIV Pathogenesis and Cure: NIH Perspectives. J. Leukoc. Biol..

[152] Wu V.H., Nordin J.M.L., Nguyen S., Joy J., Mampe F., Del Rio Estrada P.M., Torres-Ruiz F., González-Navarro M., Luna-Villalobos Y.A., Ávila-Ríos S. (2023). Profound Phenotypic and Epigenetic Heterogeneity of the HIV-1-Infected CD4+ T Cell Reservoir. Nat. Immunol..

[153] Mitchell B.I., Laws E.I., Ndhlovu L.C. (2019). Impact of Myeloid Reservoirs in HIV Cure Trials. Curr. HIV/AIDS Rep..

[154] Denton P.W., Søgaard O.S., Tolstrup M. (2019). Impacts of HIV Cure Interventions on Viral Reservoirs in Tissues. Front. Microbiol..

[155] Blackard J.T. (2012). HIV Compartmentalization: A Review on a Clinically Important Phenomenon. Curr. HIV Res..

[156] Ndhlovu Z., Moodley M., Hossain T., Chasara C., Khaba T., Mahlobo B., Reddy N., Reddy K., Ngubane T., Pansegrouw J. (2026). IBA-1+CD68+ Germinal Center Macrophages Harbor Proviral and Inducible Clade C HIV Reservoirs in ART-Suppressed Human Lymph Nodes. Res. Sq..

[157] Smith P.D., Meng G., Salazar-Gonzalez J.F., Shaw G.M. (2003). Macrophage HIV-1 Infection and the Gastrointestinal Tract Reservoir. J. Leukoc. Biol..

[158] Morón-López S., Puertas M.C., Gálvez C., Navarro J., Carrasco A., Esteve M., Manyé J., Crespo M., Salgado M., Martinez-Picado J. (2017). Sensitive Quantification of the HIV-1 Reservoir in Gut-Associated Lymphoid Tissue. PLoS ONE.

[159] Gupta R.K., Peppa D., Hill A.L., Gálvez C., Salgado M., Pace M., McCoy L.E., Griffith S.A., Thornhill J., Alrubayyi A. (2020). Evidence for HIV-1 Cure after CCR5Δ32/Δ32 Allogeneic Haemopoietic Stem-Cell Transplantation 30 Months Post Analytical Treatment Interruption: A Case Report. Lancet HIV.

[160] Trapecar M., Khan S., Roan N.R., Chen T.-H., Telwatte S., Deswal M., Pao M., Somsouk M., Deeks S.G., Hunt P.W. (2017). An Optimized and Validated Method for Isolation and Characterization of Lymphocytes from HIV+ Human Gut Biopsies. AIDS Res. Hum. Retroviruses.

[161] Mehraj V., Ghali P., Ramendra R., Costiniuk C., Lebouché B., Ponte R., Reinhard R., Sousa J., Chomont N., Cohen E.A. (2017). The Evaluation of Risk-Benefit Ratio for Gut Tissue Sampling in HIV Cure Research. J. Virus Erad..

[162] Bjerg Christensen A., Dige A., Vad-Nielsen J., Brinkmann C.R., Bendix M., Østergaard L., Tolstrup M., Søgaard O.S., Rasmussen T.A., Randel Nyengaard J. (2015). Administration of Panobinostat Is Associated with Increased IL-17A mRNA in the Intestinal Epithelium of HIV-1 Patients. Mediat. Inflamm..

[163] Elliott J.H., Wightman F., Solomon A., Ghneim K., Ahlers J., Cameron M.J., Smith M.Z., Spelman T., McMahon J., Velayudham P. (2014). Activation of HIV Transcription with Short-Course Vorinostat in HIV-Infected Patients on Suppressive Antiretroviral Therapy. PLoS Pathog..

[164] Krarup A.R., Abdel-Mohsen M., Schleimann M.H., Vibholm L., Engen P.A., Dige A., Wittig B., Schmidt M., Green S.J., Naqib A. (2018). The TLR9 Agonist MGN1703 Triggers a Potent Type I Interferon Response in the Sigmoid Colon. Mucosal Immunol..

[165] Baughman R. (2007). Technical Aspects of Bronchoalveolar Lavage: Recommendations for a Standard Procedure. Semin. Respir. Crit. Care Med..

[166] Costiniuk C.T., Jenabian M.-A. (2014). The Lungs as Anatomical Reservoirs of HIV Infection: The Lungs as HIV Reservoirs. Rev. Med. Virol..

[167] Wu V.H., Nordin J.M.L., Pampena M.B., Rachimi S., Burgess W.L., Clark M., Robinson J.A., Estrada P.M.D.R., Schleimann M.H., Torres-Ruiz F. (2025). Selective Infection and Loss of PRDM1+ LN Tfh Cells in Uncontrolled HIV Infection Precludes Formation of Tfh Reservoirs under ART 2025. bioRxiv.

[168] Chintanaphol M., Sacdalan C., Chottanapund S., Pinyakorn S., Buranapraditkun S., Crowell T.A., Kroon E., Manasnayakorn S., Chipman J.G., Schacker T.W. (2018). Brief Report: Safety and Tolerability of Inguinal Lymph Node Biopsy in Individuals With Acute HIV Infection in Thailand. JAIDS J. Acquir. Immune Defic. Syndr..

[169] Vibholm L.K., Lorenzi J.C.C., Pai J.A., Cohen Y.Z., Oliveira T.Y., Barton J.P., Garcia Noceda M., Lu C.-L., Ablanedo-Terrazas Y., Del Rio Estrada P.M. (2019). Characterization of Intact Proviruses in Blood and Lymph Node from HIV-Infected Individuals Undergoing Analytical Treatment Interruption. J. Virol..

[170] Kandathil A.J., Sugawara S., Goyal A., Durand C.M., Quinn J., Sachithanandham J., Cameron A.M., Bailey J.R., Perelson A.S., Balagopal A. (2018). No Recovery of Replication-Competent HIV-1 from Human Liver Macrophages. J. Clin. Investig..

[171] Rasmussen T.A., Tolstrup M., Brinkmann C.R., Olesen R., Erikstrup C., Solomon A., Winckelmann A., Palmer S., Dinarello C., Buzon M. (2014). Panobinostat, a Histone Deacetylase Inhibitor, for Latent-Virus Reactivation in HIV-Infected Patients on Suppressive Antiretroviral Therapy: A Phase 1/2, Single Group, Clinical Trial. Lancet HIV.

[172] Deleage C., Chan C.N., Busman-Sahay K., Estes J.D. (2018). Next-Generation in Situ Hybridization Approaches to Define and Quantify HIV and SIV Reservoirs in Tissue Microenvironments. Retrovirology.

[173] Zaman F., Smith M.L., Balagopal A., Durand C.M., Redd A.D., Tobian A.A.R. (2024). Spatial Technologies to Evaluate the HIV-1 Reservoir and Its Microenvironment in the Lymph Node. mBio.

[174] Hu K., O’Neil T.R., Baharlou H., Austin P.J., Karrasch J.F., Sarkawt L., Li Y., Bertram K.M., Cunningham A.L., Patrick E. (2025). The Spatial Biology of HIV Infection. PLoS Pathog..

[175] Mbhele N., Chimukangara B., Tyers L., Maldarelli F., Redd A.D. (2025). Advancements in Single-Cell Techniques for Examining the HIV Reservoir: Pathways to a Cure. mBio.

[176] Frouard J., Telwatte S., Luo X., Elphick N., Thomas R., Arneson D., Roychoudhury P., Butte A.J., Wong J.K., Hoh R. (2024). HIV-SEQ Reveals Global Host Gene Expression Differences Between HIV-Transcribing Cells from Viremic and Suppressed People with HIV. bioRxiv.

[177] Collora J.A., Liu R., Pinto-Santini D., Ravindra N., Ganoza C., Lama J.R., Alfaro R., Chiarella J., Spudich S., Mounzer K. (2022). Single-Cell Multiomics Reveals Persistence of HIV-1 in Expanded Cytotoxic T Cell Clones. Immunity.

[178] Wei Y., Davenport T.C., Collora J.A., Ma H.K., Pinto-Santini D., Lama J., Alfaro R., Duerr A., Ho Y.-C. (2023). Single-Cell Epigenetic, Transcriptional, and Protein Profiling of Latent and Active HIV-1 Reservoir Revealed That IKZF3 Promotes HIV-1 Persistence. Immunity.

[179] Wong M., Wei Y., Ho Y.-C. (2023). Single-Cell Multiomic Understanding of HIV-1 Reservoir at Epigenetic, Transcriptional, and Protein Levels. Curr. Opin. HIV AIDS.

